# Design of a tetravalent dengue vaccine incorporating T-cell epitopes from NS3 and NS5 proteins and B-cell epitopes from the envelope protein using an immunoinformatics approach

**DOI:** 10.3389/fcimb.2026.1934834

**Published:** 2026-10-07

**Authors:** Theerawat Dobutr, Pemikar Srifa, Nawamin Pinpathomrat

**Affiliations:** Department of Biomedical Sciences and Biomedical Engineering, Faculty of Medicine, Prince of Songkla University, Hat Yai, Songkhla, Thailand

**Keywords:** dengue virus, epitope prediction, HLA, immunoinformatics, multi-epitope vaccine

## Abstract

Dengue virus remains a major global health threat due to its four antigenically diverse serotypes and limited treatment and vaccine options. This study aimed to design and evaluate a rational tetravalent multi-epitope vaccine capable of eliciting broad cross-serotype immune responses against dengue virus. An immunoinformatics-driven strategy was employed to identify CTL, HTL, and B-cell epitopes from the NS3, NS5, and envelope domain III proteins of DENV-1 to DENV-4 based on antigenicity, allergenicity, and predicted immunogenic potential, followed by assessment of HLA population coverage. Among the selected CTL epitopes, three were completely conserved across all four serotypes, while one HTL epitope showed the highest conservation among the selected MHC class II epitopes. High-scoring linear B-cell epitopes were identified across EDIII of all four DENV serotypes, with several closely matching experimentally validated epitopes and predominantly localizing to the FG loop and DIII–DI hinge regions. The combined MHC class I and II population coverage reached 98.05% worldwide. The final vaccine construct comprised 584 amino acids incorporating 11 CTL, 14 HTL, and 7 B-cell epitopes linked to an N-terminal β-defensin adjuvant. Molecular docking, MM/GBSA calculations, and 200 ns molecular dynamics simulations demonstrated stable interactions with both TLR2 and TLR4/MD2 complexes, with docking binding energies of -16.2 and -26.0 kcal/mol and MM/GBSA binding free energies of -27.15 ± 0.54 and -52.50 ± 0.55 kcal/mol, respectively. *In silico* immune simulation predicted substantial lymphocyte activation, enhanced IFN-γ secretion, predicted antigen clearance, and immunoglobulin class switching. Collectively, the proposed tetravalent multi-epitope vaccine was predicted to provide broad cross-serotype epitope representation, substantial population coverage, and favorable structural and immunological profiles, supporting its potential for further vaccine development. Experimental studies in appropriate *in vitro* and *in vivo* models are warranted to further assess its immunogenicity, safety, and protective potential.

## 1 Introduction

Dengue fever is a mosquito-borne viral disease caused by the dengue virus, a positive-sense single-stranded RNA virus in the genus *Flavivirus* (family *Flaviviridae*) (Guzman and Harris, 2015). It is primarily transmitted by *Aedes aegypti* mosquitoes and represents one of the most significant vector-borne diseases in tropical and subtropical regions (World Health Organization (WHO), 2024). In recent decades, the global burden of dengue has increased markedly, with its geographic distribution expanding from a limited number of endemic countries to more than 100 today (World Health Organization, 2025). Although early 2026 reports indicate over 500,000 cases and more than 100 related deaths globally, representing a decrease compared to the same period in 2025, transmission remains substantial worldwide (European Centre for Disease Prevention and Control (ECDC), 2026).

Currently, no specific antiviral treatment for dengue has been approved. As a result, clinical management remains primarily supportive, focusing on careful fluid management and monitoring to prevent severe complications (Dong et al., 2025; Jiang et al., 2025). In parallel, multiple vaccine strategies for dengue have been actively developed, including live attenuated, inactivated, recombinant subunit, viral vector-based, and nucleic acid platforms (Anumanthan et al., 2025; Bello et al., 2025). However, challenges related to efficacy, safety, and the induction of balanced immune responses against all four serotypes remain unresolved (Estofolete et al., 2026).

Efforts to develop dengue vaccines have progressed substantially over recent decades, leading to several tetravalent vaccine candidates targeting all four dengue virus serotypes. Currently, Dengvaxia and TAK-003 have been licensed in multiple countries, demonstrating overall efficacies ranging from approximately 60–80% in reducing symptomatic dengue, hospitalization, and severe disease (Escudero et al., 2026; Estofolete et al., 2026; Swaminathan and Khanna, 2019). Meanwhile, Butantan-DV remains under phase 3 clinical evaluation, with reported long-term efficacy of approximately 65% over five years (Kallás et al., 2026). Despite these advances, challenges related to serotype-specific protection, durability of immunity, and safety in seronegative individuals continue to limit the development of universally effective dengue vaccines. The development of effective dengue vaccines is complicated by the need to generate balanced immune responses against all four dengue virus serotypes (Yan et al., 2025). Immunity induced by prior infection or vaccination does not always provide consistent cross-protection and may influence disease severity (Aguiar and Stollenwerk, 2020). In addition to robust and durable antibody responses, effective viral control also depends on coordinated cellular immunity (Kumbhare et al., 2025). A further challenge in dengue vaccine development is antibody-dependent enhancement (ADE), a phenomenon in which pre-existing, sub-neutralizing antibodies facilitate viral entry into Fc receptor–bearing cells, leading to increased viral replication and a higher risk of severe disease (Mersha et al., 2024; Wang et al., 2025). This mechanism has important implications for vaccine design, as incomplete or unbalanced immunity across the four dengue virus serotypes may increase the risk of severe outcomes during subsequent heterotypic infection (Halstead, 2024; Screaton et al., 2015). Although the envelope (E) protein has been the primary target for dengue vaccine development, not all regions of this protein are equally suitable for eliciting protective immunity (Yang et al., 2022). Structurally, the E protein comprises three domains (EDI, EDII, and EDIII), which differ in their immunological properties. Antibodies directed against domain III (EDIII) are generally associated with serotype-specific and strongly neutralizing activity (Medina-Carrasco et al., 2023; Williams et al., 2012). In contrast, responses targeting domains I and II, particularly the highly conserved fusion loop within EDII, tend to be cross-reactive but often weakly neutralizing (Shukla et al., 2020).

In response to these challenges, alternative viral targets have been explored. Non-structural proteins of the dengue virus, particularly NS3 and NS5, have gained attention due to their roles in viral replication and their potential as therapeutic and vaccine targets (Ahmed et al., 2020). In addition to their functional importance, these proteins contain epitopes that are processed and presented by human MHC class I molecules, enabling the activation of virus-specific CD8^+^ T cells and contributing to cellular immune responses against dengue virus infection (Jaiswal and Kushawaha, 2025). Evidence indicates that cellular immune responses are preferentially directed toward several dengue viral proteins, including NS3, capsid, NS5, and NS4A/B, underscoring their importance in shaping effective T cell–mediated immunity (Waickman et al., 2019). Several studies also considered host HLA-associated immunopathological susceptibility during epitope screening. Accumulating evidence suggests that several HLA alleles are associated with differential susceptibility to severe dengue manifestations (Sharma et al., 2023). Alleles such as HLA-A*24, HLA-A*03, HLA-A*31, HLA-B*51, and HLA-DRB1*08 have been associated with increased risk of DHF/DSS, whereas HLA-A*33, HLA-B*44, and HLA-DRB1*09:01 appear to exert protective effects against severe disease progression (Ghosh et al., 2025; Gupta et al., 2018; Malavige et al., 2011; Nguyen et al., 2008). These findings suggest that the immunological outcome of dengue infection may depend not only on viral antigen composition but also on HLA-restricted presentation of specific epitopes capable of driving either protective or pathogenic immune responses.

In recent years, immunoinformatics has emerged as a powerful interdisciplinary approach integrating bioinformatics, immunology, and computational biology to accelerate vaccine design (Basmenj et al., 2025; Patronov and Doytchinova, 2013). Given the complexity of immune system interactions and the large volume of functional, clinical, and epidemiological data, computational methods such as immunogenomics, immunoproteomics, epitope prediction, and *in silico* vaccination have become essential tools (Martinelli, 2022; Tomar and De, 2014). Immunoinformatics enables the identification and prioritization of conserved and immunologically relevant epitopes, facilitating the development of multi-epitope vaccine constructs capable of inducing both humoral and cellular immune responses (Li et al., 2026; Patronov and Doytchinova, 2013). By focusing on specific B-cell and T-cell epitopes, including HLA-restricted and cytotoxic T lymphocyte (CTL) epitopes, this approach allows precise control over antigen composition while reducing the inclusion of regions associated with suboptimal or potentially deleterious immune responses (Khan et al., 2023; Raveshi et al., 2026). Such targeted design is particularly advantageous in dengue, where balanced immunity across serotypes is critical, and supports more efficient vaccine development through *in silico* prioritization of candidate epitopes (Kaushik et al., 2022).

Despite the availability of tetravalent dengue vaccines, the identification of appropriate epitopes for broad and balanced immune protection remains challenging. Strategies focused primarily on conserved regions may overlook highly immunogenic epitopes or antigenic sites relevant to controlling dengue virus infection. Furthermore, HLA diversity and alleles reported to be associated with antibody-dependent enhancement (ADE) or dengue susceptibility warrant consideration when developing vaccines for genetically diverse populations. To address these challenges, this study employed a rational immunoinformatics-driven approach to design a tetravalent multi-epitope vaccine incorporating T-cell epitopes from NS3 and NS5 and B-cell epitopes from envelope domain III (EDIII), while considering global HLA population coverage and excluding alleles reported to be associated with ADE or dengue susceptibility. The resulting construct was subsequently evaluated for its predicted immunogenicity, structural stability, and population coverage as a potential dengue vaccine candidate.

## 2 Materials and methods

### 2.1 Sequence retrieval and alignment analysis of dengue virus E protein, NS3, and NS5

In this study, protein sequences of the dengue virus envelope (E) protein, NS3, and NS5 from all four serotypes (DENV-1: P33478, DENV-2: P14340, DENV-3: P27915, and DENV-4: P09866) were retrieved from the UniProt database, as shown in Supplementary File S1. Multiple sequence alignment (MSA) was performed using Clustal Omega available at the European Bioinformatics Institute (https://www.ebi.ac.uk/jdispatcher/msa/clustalo) (accessed on November 7, 2025) with default parameters. The resulting alignment was used to generate the phylogenetic tree, which was subsequently visualized using the Interactive Tree of Life (iTOL) platform (https://itol.embl.de) (accessed on November 7, 2025).

### 2.2 Prediction of CTL and HTL epitopes

The amino acid sequences of NS3 and NS5 proteins were subjected to immunoinformatic analysis to identify potential T-cell epitopes. For cytotoxic T lymphocyte (CTL) epitope prediction, the NetMHC 4.0 server (https://services.healthtech.dtu.dk/services/NetMHC-4.0/) was employed to estimate the binding affinity of peptides to MHC class I molecules (Andreatta and Nielsen, 2016) (**Figure 1D**). A diverse set of HLA class I alleles, including HLA-A*33:01, HLA-A*01:01, HLA-A*02:01, HLA-B*07:02, HLA-B*15:01, HLA-B*40:01, HLA-B*58:01, HLA-A*26:01, HLA-B*08:01, HLA-B*27:05, HLA-B*39:01, HLA-B*44:02, and HLA-B*44:03, was included in the analysis.

**Figure 1:**
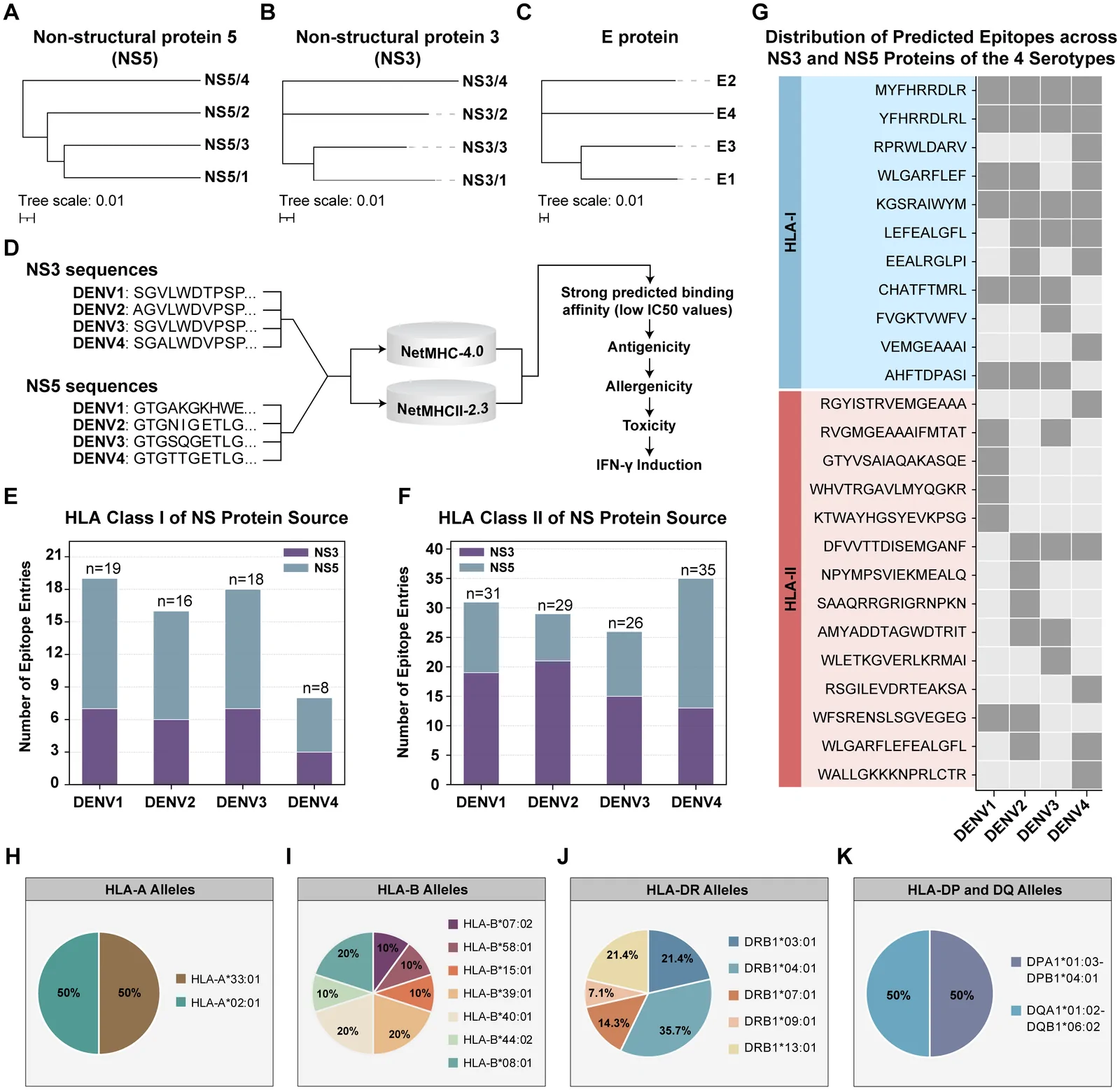
Phylogenetic analysis and T-cell epitope prediction across the four dengue virus serotypes (DENV1–4). **(A–C)** Phylogenetic trees of NS5, NS3, and the envelope (E) protein. **(D)** Workflow for MHC class I and II T-cell epitope prediction and selection based on binding affinity, antigenicity, allergenicity, toxicity, and IFN-γ induction. **(E, F)** Distribution of predicted HLA class I and II epitopes derived from NS3 and NS5 across DENV1–4. **(G)** Distribution of HLA class I and II epitope candidates across dengue serotypes. **(H–K)** Selected epitopes associated with HLA-A, HLA-B, HLA-DR, and HLA-DP/DQ alleles.

For helper T lymphocyte (HTL) epitope prediction, the NetMHCII 2.3 server (https://services.healthtech.dtu.dk/services/NetMHCII-2.3/) was utilized to evaluate peptide binding to MHC class II molecules (Jensen et al., 2018). The selected alleles included HLA-DRB1*04:01, HLA-DRB1*07:01, HLA-DRB1*09:01, HLA-DRB1*11:01, HLA-DRB1*13:01, HLA-DRB1*15:01, and HLA-DRB1*03:01, along with HLA-DQ and HLA-DP combinations (HLA-DQA1*01:01-DQB1*05:01, HLA-DQA1*01:02-DQB1*06:02, HLA-DQA1*03:01-DQB1*03:02, HLA-DPA1*02:01-DPB1*14:01, HLA-DPA1*01:03-DPB1*04:01, and HLA-DPA1*03:01-DPB1*04:02).

Candidate epitopes were subsequently selected based on strong predicted binding affinity, as indicated by low IC_50_ values and favorable percentile rankings. In addition, the selected epitopes were further evaluated for their ability to induce interferon-gamma (IFN-γ) responses using the IFNepitope server (http://crdd.osdd.net/raghava/ifnepitope/).

### 2.3 Structure-based prediction and mapping of B-cell epitopes

The amino acid sequences of the E protein from DENV-1 to DENV-4 were retrieved from the UniProt database, as described in Section 2.1. Homology models of the E proteins were generated using the SWISS-MODEL server (https://swissmodel.expasy.org) based on experimentally determined homologous structural templates. The resulting three-dimensional structures were obtained as PDB files and used as input for conformational B-cell epitope prediction using the ElliPro server (https://tools.iedb.org/ellipro/) (Ponomarenko et al., 2008). ElliPro identifies potential antibody-accessible regions based on the three-dimensional structure of the protein. Regions with high protrusion index (PI) scores, indicative of surface-exposed and protruding regions, were prioritized. Particular emphasis was placed on domain III (DIII) of the E protein from each serotype because of its established involvement in receptor binding and immunogenicity (**Figures 2A, B**).

**Figure 2:**
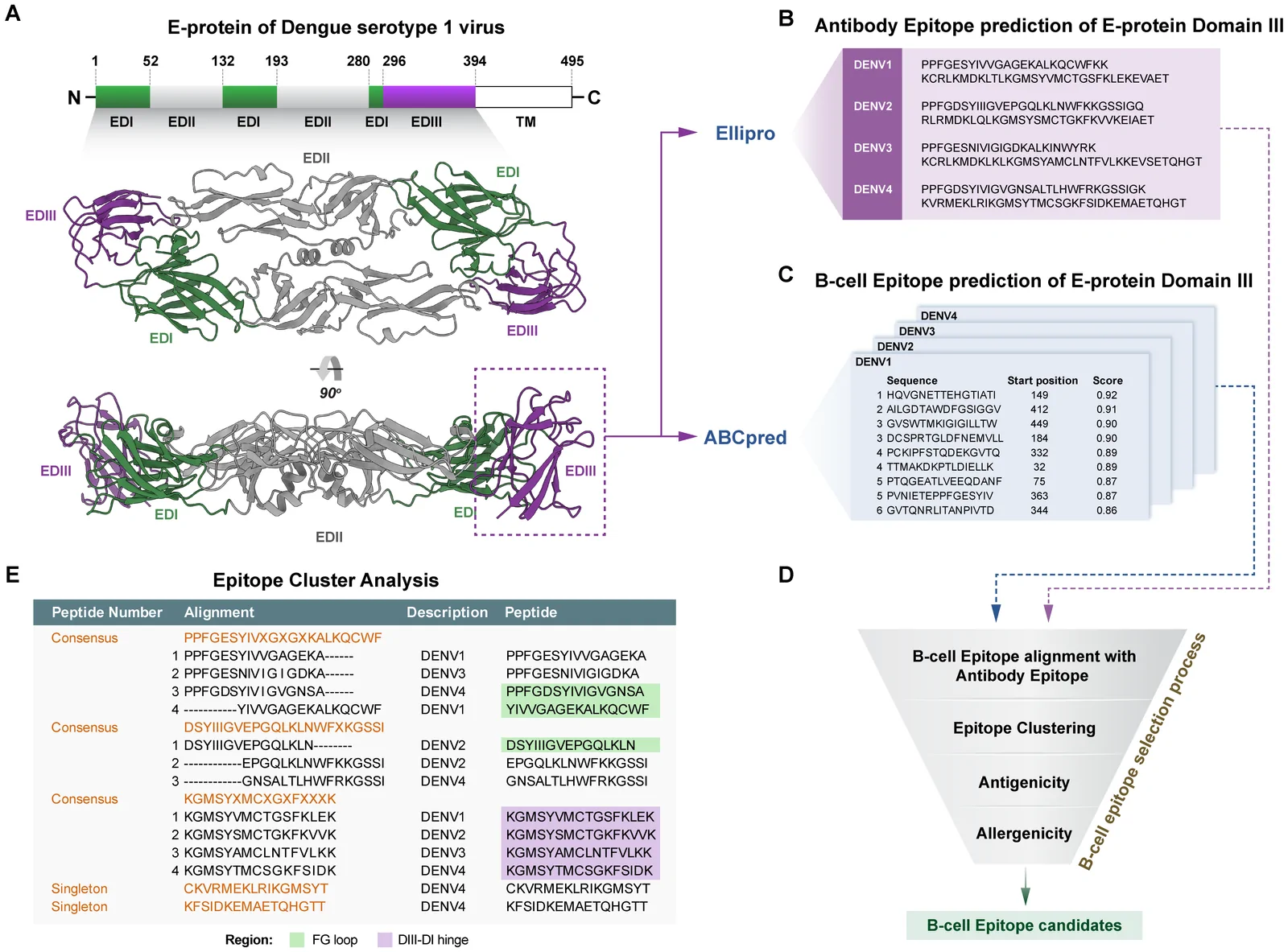
Identification and selection of B-cell epitopes from dengue virus envelope protein domain III (EDIII). **(A)** Domain organization and structure of the E protein showing EDI, EDII, and EDIII. **(B)** Structure-based antibody epitope prediction of EDIII sequences from DENV1–4 using ElliPro. **(C)** Linear B-cell epitope prediction using ABCpred. **(D)** B-cell epitope selection workflow based on epitope overlap, clustering, antigenicity, allergenicity, and toxicity. **(E)** Epitope clusters showing conserved and serotype-specific B-cell epitope candidates across DENV1–4.

Subsequently, linear B-cell epitopes were predicted using the ABCpred server (http://crdd.osdd.net/raghava/abcpred/) (Saha and Raghava, 2006), which applies an artificial neural network-based approach. The predicted linear epitopes were then mapped onto the structurally accessible regions identified by Ellipro. Only epitopes that overlapped between the two methods were selected, ensuring both structural accessibility and sequence-based antigenic potential (**Figures 2C, D**).

### 2.4 *In silico* assessment of antigenicity, allergenicity, and toxicity of predicted epitopes

To select suitable epitope candidates, the predicted peptides were evaluated based on their immunogenicity and safety profiles. Antigenicity was first assessed using the VaxiJen v2.0 server (threshold: 0.4) (https://www.ddg-pharmfac.net/vaxijen/VaxiJen/VaxiJen.html) (Doytchinova and Flower, 2007), an alignment-independent method based on the physicochemical properties of proteins. Allergenicity was then evaluated using the AllerTOP v2.1 server (https://www.ddg-pharmfac.net/allertop_test/), and only epitopes classified as probable non-allergen were selected for further analysis. Finally, toxicity prediction was performed using the ToxinPred server (http://crdd.osdd.net/raghava/toxinpred/) and only non-toxic peptides were considered (Gupta et al., 2013). Epitopes that met all predefined criteria were included in subsequent analyses.

### 2.5 Multi-epitope vaccine construction

The final multi-epitope vaccine construct was designed by integrating selected CTL, HTL, and B-cell epitopes into a single chimeric sequence. An immunostimulatory adjuvant, β-defensin (UniProt ID: Q5U7J2), was incorporated at the N-terminus to promote immune activation and enhance both innate and adaptive immune responses (Chi et al., 2024). The adjuvant was linked to the downstream epitopes using the EAAK linker to provide structural rigidity and to minimize potential interference between functional domains.

Cytotoxic T lymphocyte (CTL) epitopes were arranged sequentially and joined using AAY linkers, which facilitate efficient proteasomal processing and epitope presentation via MHC class I molecules. Subsequently, helper T lymphocyte (HTL) epitopes were incorporated and connected using GPGPG linkers to promote proper processing and presentation through MHC class II pathways while reducing junctional immunogenicity.

Finally, B-cell epitopes were appended at the C-terminus of the construct and linked using KK linkers to maintain epitope integrity and enhance accessibility for antibody recognition. The overall construct was designed to ensure optimal epitope presentation, structural stability, and immunogenic potential for downstream vaccine development.

### 2.6 *In silico* evaluation of multi-epitope vaccine construct

The physicochemical and structural properties of the designed multi-epitope vaccine construct were comprehensively evaluated using established bioinformatics tools. The presence of transmembrane helices and overall membrane protein topology were predicted using the TMHMM 2.0 server (https://services.healthtech.dtu.dk/services/TMHMM-2.0/) (Krogh et al., 2001) to ensure the construct does not contain undesirable transmembrane regions that may affect expression and stability. Physicochemical characteristics of the vaccine construct, including molecular weight, theoretical isoelectric point (pI), instability index, aliphatic index, and grand average of hydropathicity (GRAVY), were computed using the ProtParam tool (https://web.expasy.org/protparam/) to assess its stability and suitability for downstream applications (Wilkins et al., 1999). Protein solubility was predicted using the Protein-Sol server (https://protein-sol.manchester.ac.uk/) based on experimental solubility data, where values >0.45 indicate higher-than-average solubility in *E*. *coli* (Hebditch et al., 2017).

Furthermore, the immunological and safety profiles of the construct, including antigenicity, allergenicity, and toxicity, were evaluated as described previously, confirming its suitability for vaccine development.

### 2.7 3D structural prediction and validation of the vaccine construct

The 3D structure of the designed multi-epitope vaccine construct was predicted using the AlphaFold server (https://alphafoldserver.com) (Abramson et al., 2024). The predicted model was evaluated for structural reliability using the Predicted Aligned Error (PAE) using PAE Viewer (https://pae-viewer.uni-goettingen.de) to assess local confidence and domain organization (Elfmann and Stülke, 2023).

Further structural validation was performed using the ProSA-web server (https://prosa.services.came.sbg.ac.at/prosa.php) to determine the overall model quality based on the Z-score, indicating the compatibility of the predicted structure with experimentally determined protein structures (Wiederstein and Sippl, 2007). In addition, stereochemical quality was assessed using Ramachandran plot analysis (https://www.ramplot.in) to evaluate the distribution of backbone dihedral angles (φ and ψ), ensuring that residues are predominantly located within favored regions (Kumar and Rathore, 2025).

### 2.8 Molecular docking with immune receptors

To investigate the interaction between the vaccine construct and innate immune receptors, molecular docking analysis was performed with Toll-like receptor 2 (TLR2) and Toll-like receptor 4 (TLR4)/myeloid differentiation factor 2 (MD2). The 3D structures of TLR2 (PDB ID: 2Z7X) and TLR4/MD2 (PDB ID: 4G8A) were retrieved from the Protein Data Bank database. Prior to docking, the protein structures were prepared using the Dock Prep tool implemented in UCSF ChimeraX to optimize the structures by removing solvent molecules and non-essential heteroatoms, adding hydrogen atoms, assigning atomic charges, and correcting incomplete side chains where necessary (Meng et al., 2023). Protein-protein docking was subsequently conducted using HADDOCK 2.4 (https://wenmr.science.uu.nl/haddock2.4/) (Honorato et al., 2024). The vaccine construct was docked against TLR2 and TLR4 to predict binding interactions and identify the most favorable complexes.

The docked complexes were ranked based on docking score, confidence score, and ligand root mean square deviation (RMSD). The top-ranked complexes were subsequently analyzed using the PRODIGY server (https://wenmr.science.uu.nl/prodigy/manual) to estimate binding affinity (ΔG) and dissociation constant (Kd), providing further insight into the stability and strength of the interactions (Xue et al., 2016).

### 2.9 Molecular dynamics simulation

Molecular dynamics (MD) simulations were performed using GROMACS version 2025.3 (Berendsen et al., 1995) with the AMBER99SB-ILDN force field and the TIP3P water model. Four systems were prepared, including TLR2-DENVAC/4, TLR4-DENVAC/4, TLR2 apo, and TLR4 apo. Each system was placed in a cubic simulation box with a minimum distance of 1.0 nm between the protein and the box edges. All systems were solvated with TIP3P water molecules and neutralized by the addition of Na^+^ and Cl^-^ ions to achieve a final ionic concentration of 0.15 M. Energy minimization was carried out using the steepest descent algorithm until the maximum force was below 1,000 kJ/mol/nm. The systems were then equilibrated under NVT conditions at 300 K for 100 ps using a V-rescale thermostat, followed by NPT equilibration at 300 K and 1.0 bar for 100 ps using a Parrinello-Rahman barostat.

Production MD simulations were subsequently conducted for 200 ns with a 2 fs time step. Long-range electrostatic interactions were treated using the Particle Mesh Ewald (PME) method with a cutoff of 1.0 nm, and all bonds involving hydrogen atoms were constrained using the LINCS algorithm. Trajectory analyses, including root mean square deviation (RMSD), root mean square fluctuation (RMSF), radius of gyration (Rg), and hydrogen bond analysis, were performed across the full 200 ns production trajectory (10–200 ns, frames 1000–20000 with an interval of 10 frames).

Binding free energy calculations were performed using the MM/GBSA (Molecular Mechanics Generalized Born Surface Area) method implemented in gmx_MMPBSA v1.5.0.3 with AmberTools 20 (Valdés-Tresanco et al., 2021). Calculations were carried out on 1,901 frames extracted from the production MD trajectories, spanning from frame 1,000 to frame 20,000 with an interval of 10 frames (10–200 ns). The Generalized Born model with igb=5 (OBC2) was employed for implicit solvation, with a salt concentration of 0.15 M. Non-polar solvation contributions were estimated using the LCPO method for solvent accessible surface area (SASA) calculations. The binding free energy was calculated using the single trajectory approach as:

ΔG_binding = G_complex − G_receptor − G_ligand

where each term comprises gas-phase energy (ΔGGAS) and solvation free energy (ΔGSOLV) contributions. Entropic contributions were not included in the calculations. For each system, the vaccine construct was defined as the ligand and the TLR protein as the receptor.

### 2.10 Computational immune simulation using C-ImmSim

The protein sequence of the multi-epitope vaccine was evaluated for its potential to elicit both cellular and humoral immune responses using the C-ImmSim web server (https://kraken.iac.rm.cnr.it/C-IMMSIM/index.php) (Rapin et al., 2011). The simulation parameters were configured with a simulation volume of 10 µL and a total of 1050 simulation steps, corresponding to approximately 350 days of immune response. To ensure reproducibility, a fixed random seed (12345) was applied throughout the simulation. The host HLA alleles selected for the analysis included HLA-A*33:01, HLA-A*02:01, HLA-B*39:01, HLA-B*44:02, HLA-DRB1*07:01, and HLA-DRB1*03:01. Three injections were administered at time steps 1, 84, and 168, representing a prime–boost immunization schedule. An adjuvant value of 1000 was applied to enhance immune stimulation. The simulation outputs were analyzed in terms of antigen concentration, immunoglobulin responses (IgM and IgG), dendritic cell, B-cell, and T-cell populations, cytokine profiles, and the generation of immunological memory.

### 2.11 Population coverage analysis

Population coverage analysis was performed to estimate the global and regional applicability of the selected CTL and HTL epitopes based on their associated HLA alleles. The analysis was conducted using the IEDB Population Coverage tool (Bui et al., 2006). A total of 11 CTL epitopes restricted to 9 HLA class I alleles, including HLA-A*02:01, HLA-A*33:01, HLA-B*07:02, HLA-B*08:01, HLA-B*15:01, HLA-B*39:01, HLA-B*40:01, HLA-B*44:02, and HLA-B*58:01, and 14 HTL epitopes restricted to 9 HLA class II alleles, including HLA-DRB1*03:01, HLA-DRB1*04:01, HLA-DRB1*07:01, HLA-DRB1*09:01, HLA-DRB1*13:01, HLA-DQA1*01:02, HLA-DQB1*06:02, HLA-DPA1*01:03, and HLA-DPB1*04:01, were used as input.

The epitope–HLA combinations were submitted to the tool to calculate population coverage across global and regional populations using HLA allele frequency data. The analysis provides the estimated fraction of individuals predicted to respond to at least one epitope, along with additional parameters including average hit and PC90 values. Default settings were applied for all calculations.

### 2.12 Codon optimization and *in silico* protein expression

Following the construction of the multi-epitope vaccine, codon optimization and *in silico* protein expression analysis were performed to evaluate its suitability for heterologous expression in *Escherichia coli*. The amino acid sequence of the designed vaccine construct was reverse-translated into a nucleotide sequence using EMBOSS Backtranseq (https://www.ebi.ac.uk/jdispatcher/st/emboss_backtranseq) and optimized using the codon optimization tool of vectorbuilder (https://en.vectorbuilder.com/tool/codon-optimization.html).

During codon optimization, specific parameters were applied to enhance expression efficiency and transcriptional stability. These included the avoidance of prokaryotic ribosome binding sites, rho-independent transcription terminators, and internal restriction enzyme cleavage sites. The optimized gene sequence was further analyzed for codon adaptation index (CAI) and GC content to assess its compatibility with the *E. coli* expression system. A CAI value close to 1.0 and a GC content within the optimal range of 30-70% were considered indicative of efficient expression.

To facilitate downstream cloning, restriction enzyme recognition sites (e.g., NdeI and XhoI) were incorporated at the 5′ and 3′ ends of the optimized gene sequence, respectively. The resulting sequence was then *in silico* cloned into the pET-28a(+) expression vector, which contains a T7 promoter system for high-level expression in *E. coli*. A 6×His-tag was included at the appropriate terminus of the construct to enable affinity purification of the recombinant protein. The final recombinant plasmid construct was analyzed and visualized using SnapGene software (www.snapgene.com) to confirm correct insertion, orientation, and reading frame integrity.

## 3 Results

### 3.1 Sequence analysis

Phylogenetic analysis of the dengue virus NS5, NS3, and envelope (E) proteins revealed a consistent pattern of low genetic divergence among all analyzed sequences (tree scale = 0.01), indicating a high degree of conservation. For the NS5 protein, sequences from DENV-1 to DENV-4 clustered closely, with DENV-1 and DENV-3 forming the most closely related pair, followed by DENV-2, while DENV-4 appeared as the most divergent within the group (**Figure 1A**). A similar trend was observed for the NS3 protein, where DENV-1 and DENV-3 grouped together as the closest pair, followed by DENV-2, and DENV-4 positioned as the most distantly related sequence (**Figure 1B**). In the E protein, DENV-1 and DENV-3 showed the highest similarity and clustered together, followed by DENV-4, whereas DENV-2 was the most divergent (**Figure 1C**; **Supplementary File S2**).

### 3.2 *In silico*-predicted T-cell epitopes and their MHC class I and II restriction profiles across DENV serotypes

Candidate epitopes were selected based on strong predicted binding affinity and IFN-γ-inducing potential, with IFNepitope prediction scores ranging from 0.0039 to 0.9319. Among the CTL epitopes, several peptides demonstrated complete conservation across all serotypes. Specifically, MYFHRRDLR (HLA-A*33:01), YFHRRDLRL (HLA-B*08:01), and KGSRAIWYM (HLA-B*58:01) showed 100% across all four DENV serotypes, indicating their strong potential as universal T-cell targets (**Table 1**, **Figure 1G**, **Supplementary File S3**). In contrast, moderately conserved epitopes such as WLGARFLEF, LEFEALGFL, CHATFTMRL, and AHFTDPASI exhibited 75% conservancy, suggesting partial cross-serotype coverage. Lower conservancy levels were observed for EEALRGLPI (50%) and several epitopes including RPRWLDARV, FVGKTVWFV, and VEMGEAAAI, each conserved in only one serotype (25%).

**Table 1:** Predicted CTL and HTL epitopes of dengue virus with corresponding HLA alleles and epitope conservancy across DENV serotypes.

Peptides	HLA Alleles	Dengue serotypes				Epitope conservancy
		1	2	3	4	
CTL epitopes						
MYFHRRDLR	HLA-A*33:01	/	/	/	/	100% (4/4)
YFHRRDLRL	HLA-B*08:01	/	/	/	/	100% (4/4)
RPRWLDARV	HLA-B*07:02				/	25% (1/4)
WLGARFLEF	HLA-B*15:01, HLA-B*08:01	/	/		/	75% (3/4)
KGSRAIWYM	HLA-B*58:01	/	/	/	/	100% (4/4)
LEFEALGFL	HLA-B*40:01		/	/	/	75% (3/4)
EEALRGLPI	HLA-B*44:02		/		/	50% (2/4)
CHATFTMRL	HLA-B*39:01	/	/	/		75% (3/4)
FVGKTVWFV	HLA-A*02:01			/		25% (1/4)
VEMGEAAAI	HLA-B*40:01				/	25% (1/4)
AHFTDPASI	HLA-B*39:01	/	/	/		75% (3/4)
HTL epitopes						
RGYISTRVEMGEAAA	DRB1*04:01				/	25% (1/4)
RVGMGEAAAIFMTAT	DQA1*01:02-DQB1*06:02	/		/		50% (2/4)
GTYVSAIAQAKASQE	DRB1*04:01	/				25% (1/4)
WHVTRGAVLMYQGKR	DRB1*07:01	/				25% (1/4)
KTWAYHGSYEVKPSG	DRB1*07:01	/				25% (1/4)
DFVVTTDISEMGANF	DRB1*04:01, DRB1*03:01		/	/	/	75% (3/4)
NPYMPSVIEKMEALQ	DRB1*09:01		/			25% (1/4)
SAAQRRGRIGRNPKN	DRB1*13:01		/			25% (1/4)
AMYADDTAGWDTRIT	DRB1*04:01, DRB1*03:01		/	/		50% (2/4)
WLETKGVERLKRMAI	DRB1*13:01			/		25% (1/4)
RSGILEVDRTEAKSA	DRB1*03:01				/	25% (1/4)
WFSRENSLSGVEGEG	DRB1*04:01	/	/			50% (2/4)
WLGARFLEFEALGFL	DPA1*01:03-DPB1*04:01		/		/	50% (2/4)
WALLGKKKNPRLCTR	DRB1*13:01				/	25% (1/4)

For HTL epitopes, only one peptide, DFVVTTDISEMGANF (binding to HLA-DRB1*04:01 and DRB1*03:01), demonstrated relatively high conservancy at 75%. A subset of epitopes, including RVGMGEAAAIFMTAT, AMYADDTAGWDTRIT, WFSRENSLSGVEGEG, and WLGARFLEFEALGFL, showed moderate conservancy (50%). The majority of HTL epitopes exhibited serotype-specific conservancy at 25% (**Supplementary File S4**).

Predicted HLA class I and class II epitopes were identified across all four DENV serotypes from both NS3 and NS5 proteins. Overall, NS5 contributed a greater proportion of epitope entries than NS3, particularly for HLA class I-associated epitopes (**Figures 1E, F**). Among the serotypes, DENV1 showed the highest number of HLA class I epitope entries, whereas DENV4 exhibited the greatest number of HLA class II-associated epitopes. Analysis of HLA allele distribution demonstrated broad epitope association across multiple HLA alleles, with notable representation of HLA-A*33:01, HLA-A*02:01, HLA-B*39:01, HLA-B*40:01, HLA-B*08:01, DRB1*04:01, DPA1*01:03-DPB1*04:01, and DQA1*01:02-DQB1*06:02 (**Figures 1H–K**).

### 3.3 Prediction and mapping of B-cell epitopes within E protein domain III

In DENV-1, two epitopes, YIVVGAGEKALKQCWF and KGMSYVMCTGSFKLEK, were identified within domain III, both exhibiting ABCpred scores of 0.83 (**Table 2**; **Figure 2E**; **Supplementary Files S5**, **S6**). Structural mapping revealed that YIVVGAGEKALKQCWF was located within the FG loop region of EDIII and showed strong similarity to the experimentally reported IEDB epitope YIVVGAGEKALKQCWFK reported by Nadugala et al. (2021). In contrast, KGMSYVMCTGSFKLEK was located at the DIII-DI hinge region, although no corresponding epitope was identified in the IEDB database. Similarly, DENV-2 showed two high-scoring epitopes, DSYIIIGVEPGQLKLN (0.87) and KGMSYSMCTGKFKVVK (0.89), indicating strong B-cell epitope potential. The epitope DSYIIIGVEPGQLKLN was mapped to the FG loop region and partially corresponded to the experimentally validated epitope EPGQLKLNWFKKGSS reported by Anandarao et al. (2005). Meanwhile, KGMSYSMCTGKFKVVK was localized within the DIII–DI hinge region and closely matched the IEDB-reported sequence KGMSYSMCTGKFKVV described by Gromowski et al., 2008.

**Table 2:** Comparison of predicted B-Cell epitope candidates with IEDB-reported epitopes across the four DENV serotypes.

Predicted B-cell epitopes	Serotypes	EDIII regions	IEDB-reported epitope sequences^a^	
			Sequences	References
YIVVGAGEKALKQCWF	DENV-1	FG loop	YIVVGAGEKALKQCWFK	(Nadugala et al., 2021)
KGMSYVMCTGSFKLEK	DENV-1	DIII-DI hinge	N/A	–
DSYIIIGVEPGQLKLN	DENV-2	FG loop	EPGQLKLNWFKKGSS	(Anandarao et al., 2005)
KGMSYSMCTGKFKVVK	DENV-2	DIII-DI hinge	KGMSYSMCTGKFKVV	(Gromowski et al., 2008)
KGMSYAMCLNTFVLKK	DENV-3	DIII-DI hinge	KGMSYAMCLNTFVLK	(Matsui et al., 2009)
PPFGDSYIVIGVGNSA	DENV-4	FG loop	FDSYIVIGVGNSALTLH	(Nadugala et al., 2021)
KGMSYTMCSGKFSIDK	DENV-4	DIII-DI hinge	N/A	–

^a^ These sequences were confirmed by positive binding assay.

Underlined sequences indicate similar amino acid sequences and N/A indicates that the data were not available.

For DENV-3, only a single epitope, KGMSYAMCLNTFVLKK (0.83), was detected within the domain III region, with no additional suitable epitopes identified in the analyzed region. This epitope was positioned within the DIII–DI hinge region and demonstrated strong similarity to the experimentally reported epitope KGMSYAMCLNTFVLK identified by Matsui et al. (2009). DENV-4 exhibited two predicted epitopes within the same domain, namely PPFGDSYIVIGVGNSA (0.79) and KGMSYTMCSGKFSIDK (0.85). The epitope PPFGDSYIVIGVGNSA was located within the FG loop region and showed partial similarity to the reported IEDB epitope FDSYIVIGVGNSALTLH described by Nadugala et al. (2021). KGMSYTMCSGKFSIDK was mapped to the DIII–DI hinge region, although no corresponding experimentally validated epitope was identified in the IEDB database.

### 3.4 Vaccine construct design and structural characterization

A rationally designed multi-epitope subunit vaccine, DENVAC/4, was developed to target all four DENV serotypes by incorporating selected T- and B-cell epitopes into a single construct (**Figures 3A, D**). The construct consisted of 584 amino acids, comprising an N-terminal β-defensin sequence, 25 MHC-restricted T-cell epitopes (11 CTL and 14 HTL epitopes), and seven linear B-cell epitopes derived from domain III (DIII) of the E protein. These components were assembled using AAY, GPGPG, and KK linkers (**Supplementary File S7**).

**Figure 3:**
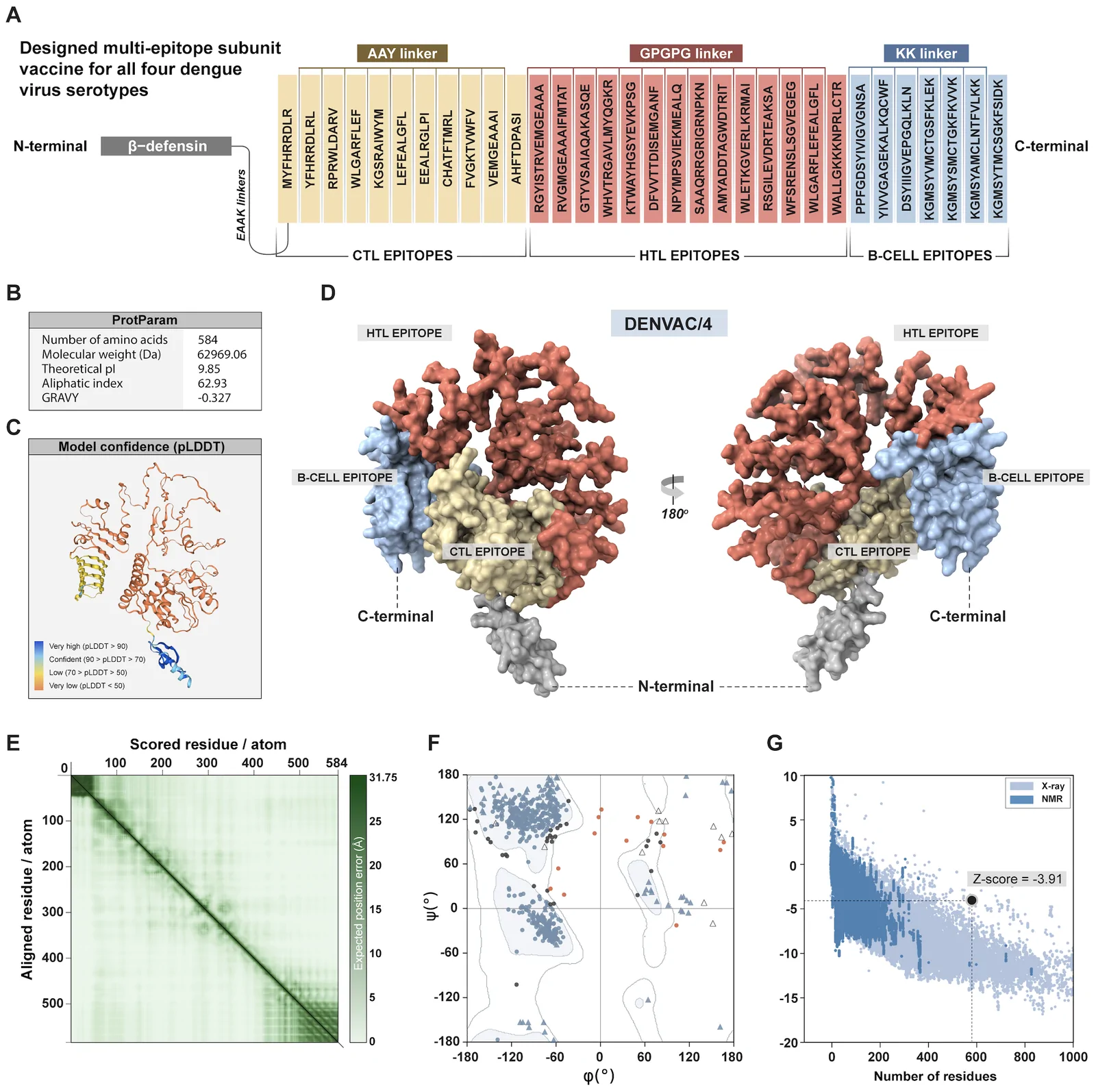
**(A)** Schematic representation of the DENVAC/4 multi-epitope vaccine construct containing CTL, HTL, and LBL epitopes with an N-terminal β-defensin adjuvant. **(B)** Predicted physicochemical properties of DENVAC/4. **(C)** Predicted 3D structure of DENVAC/4 colored according to pLDDT confidence scores. **(D)** Structural organization of DENVAC/4 showing CTL epitopes (beige), HTL epitopes (burnt red), LBL epitopes (light blue), and the adjuvant region (grey). **(E)** Predicted aligned error (PAE) matrix. **(F)** Ramachandran plot and **(G)** ProSA Z-score analysis of the predicted DENVAC/4 structure.

TMHMM analysis predicted the absence of complete transmembrane helices within the construct, with approximately 8 amino acid residues expected to be located within transmembrane regions. The complete sequence was assigned an extracellular topology, with no transmembrane region predicted within the first 67 amino acid residues. The predicted scaled solubility of the vaccine construct was 0.531, indicating favorable solubility (**Supplementary File S8**). Physicochemical analysis indicated a basic protein with a molecular weight of 62,969.06 Da, a theoretical pI of 9.85, an instability index of 31.36, and a GRAVY value of -0.327 (**Figure 3B**).

Three-dimensional structural modeling produced a compact, globular conformation with low prediction confidence across most of the construct (**Figure 3C**). The PAE analysis showed generally low error values along the diagonal, with relatively higher error in several off-diagonal regions, particularly around residues 1-50 and near the C-terminal region (**Figure 3E**). Structural validation showed that 89.69% of residues were located in favored regions of the Ramachandran plot, supporting acceptable stereochemical quality (**Figure 3F**). The modeled structure exhibited a ProSA-web Z-score of -3.91, within the range observed for experimentally determined protein structures (**Figure 3G**).

### 3.5 Molecular docking of DENVAC4 with TLR4/MD2 and TLR2

Molecular docking analysis showed that the DENVAC/4 vaccine construct was predicted to form favorable complexes with both TLR4/MD2 and TLR2, with stronger binding observed in the TLR4 system (ΔG = -26 kcal mol^-^^1^; Kd = 8.6 × 10^-^^20^ M) compared to TLR2 (ΔG = -16.2 kcal mol^-^^1^; Kd = 1.2 × 10^-^^12^ M). In the TLR4/MD2-DENVAC/4 complex (overlap cutoff -0.4 Å), interactions were distributed across multiple regions of the receptor surface, with major contributions from loop regions 158-170, 186-194, and 209-216, together with additional contacts involving MD2 (**Figure 4A**; **Supplementary File S9**). Interface mapping showed that residues involved in binding were distributed across multiple regions of the vaccine construct. Interacting residues were identified within the adjuvant region (1-45), including TYR9 and ARG12-ARG17, the CTL epitope region (50-187), including MET50, TYR51, TYR97, ARG101, TYR133, and TYR157, and the B-cell epitope region (461-584), including PHE580. In contrast, interactions with MD2 were mainly associated with residues located within the CTL region, particularly between positions TRP77 and ILE130.

**Figure 4:**
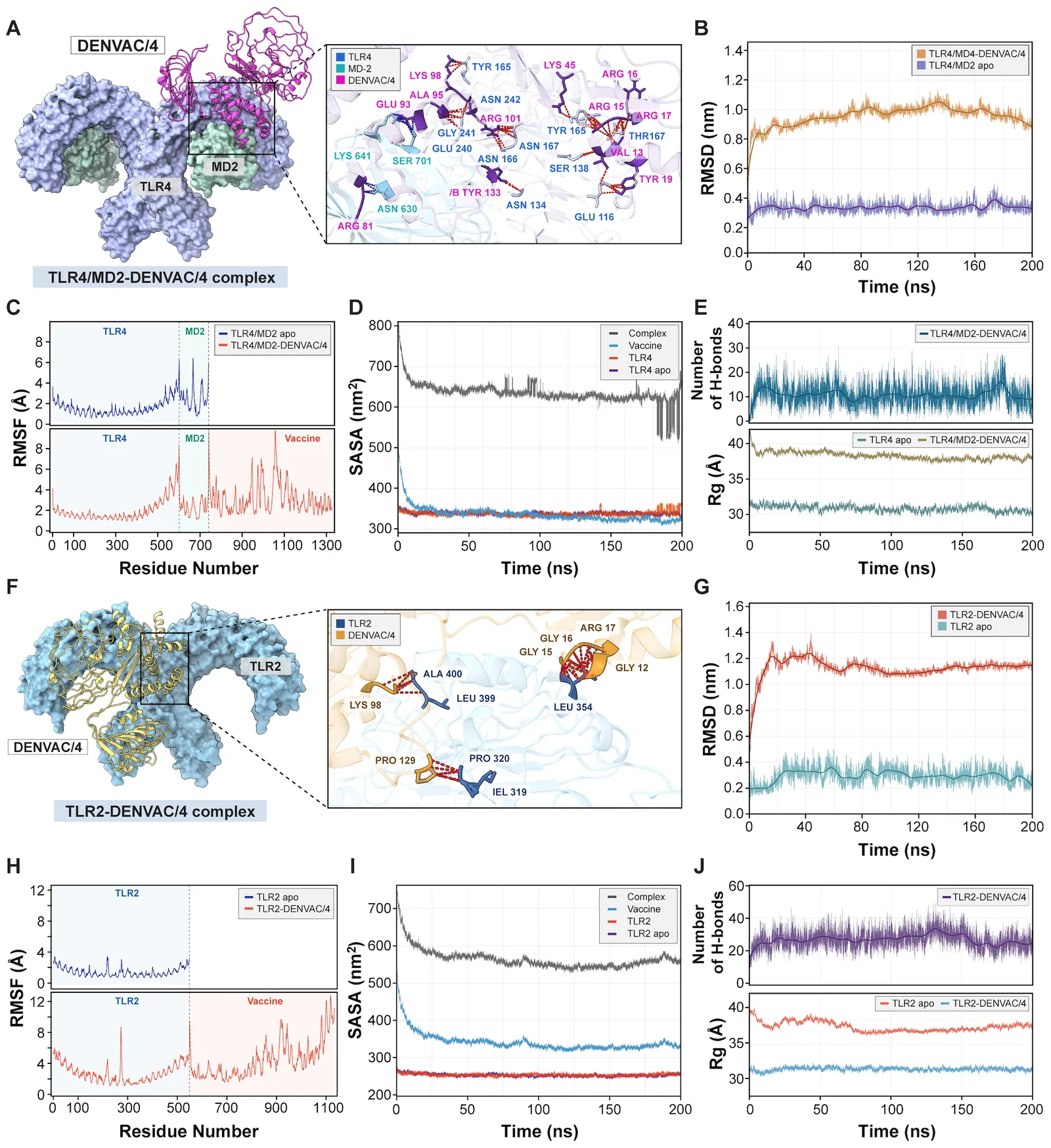
**(A, F)** Structural overviews and key interfacial interactions of the TLR4/MD2-DENVAC/4 and TLR2-DENVAC/4 complexes. **(B, G)** RMSD profiles over 200 ns for the vaccine-bound and apo systems. **(C, H)** RMSF profiles of the TLR4/MD2 and TLR2 systems in apo and vaccine-bound states. **(D, I)** SASA profiles of the complexes and individual components during simulation. **(E, J)** Intermolecular hydrogen bonds and radius of gyration (Rg) of the complexes.

In the TLR2-vaccine complex, interactions were distributed along the receptor surface, with the highest contact density observed in the core loop region (370-384), followed by the N-terminal loop (318-336) and the mid-region (396-411) (**Figure 4F**). Residue mapping indicated that binding involved residues from multiple regions of the vaccine construct. Interacting residues were identified within the adjuvant region (1-45), including TYR9 and ARG12-ARG17, and the CTL epitope region (50-187), including MET50, TYR51, TYR97, ARG101, TYR133, and TYR157. Additional interacting residues were observed in the B-cell epitope region (461-584), particularly between residues 574 and 584. In contrast, residues from the HTL region (184-458) were less represented within the major interaction clusters.

### 3.6 Molecular dynamics simulation analysis

The RMSD profiles showed that both complexes reached equilibrium after the initial phase of the simulation. The TLR4/MD2-DENVAC/4 complex stabilized after approximately 20 ns, with an average RMSD of approximately 1.0 nm, whereas the TLR2-DENVAC/4 complex stabilized at approximately 1.1-1.2 nm following initial fluctuations (**Figures 4B, G**). RMSF analysis further indicated that the observed fluctuations were primarily localized to flexible regions, particularly within the vaccine component and terminal or loop-exposed regions, while the structured receptor domains remained comparatively stable throughout the simulations (**Figures 4C, H**).

In addition, the SASA profiles remained relatively stable throughout the simulations, indicating no major changes in the overall solvent exposure of either complex (**Figures 4D, I**). Persistent intermolecular hydrogen bonds were also observed in both systems, with approximately 10–25 hydrogen bonds in the TLR4/MD2-DENVAC/4 complex and 20–40 hydrogen bonds in the TLR2-DENVAC/4 complex (**Figures 4E, J**, upper panels). Furthermore, the radius of gyration remained relatively constant for both complexes, indicating that their overall structural compactness was maintained throughout the simulations (**Figures 4E, J**, lower panels).

To further assess the interaction stability, MM/GBSA analysis was performed. Electrostatic and van der Waals interactions contributed favorably to complex formation, whereas polar solvation energy opposed binding (**Table 3**). The calculated binding free energies were -52.50 ± 0.55 kcal/mol for the TLR4/MD2-DENVAC/4 complex and -27.15 ± 0.54 kcal/mol for the TLR2-DENVAC/4 complex, indicating a more favorable predicted binding affinity of DENVAC/4 for TLR4/MD2.

**Table 3:** MM/GBSA binding free energy analysis of TLR2-DENVAC/4 and TLR4/MD2- DENVAC/4 complexes.

Energy component (kcal/mol)	TLR2-DENVAC/4 complex	TLR4/MD2-DENVAC/4 complex
ΔVDWAALS	-74.64	-139.43
ΔEEL	-1026.89	-3387.53
ΔGGAS	-1101.53	-3526.96
ΔEGB/EPB	1085.17	3493.58
ΔESURF/ENPOLAR	-10.79	-19.13
ΔGSOLV	1074.38	3474.45
ΔTOTAL	-27.15 ± 0.54	-52.50 ± 0.55

### 3.7 *In silico* evaluation of vaccine-induced immune responses

C-ImmSim immune simulation demonstrated sustained innate and adaptive immune responses throughout the 350-day observation period. Total dendritic cell populations remained relatively stable at 150–250 cells/mm³, with resting dendritic cells predominating, while active dendritic cells remained at low levels throughout the simulation (**Figure 5A**). Active helper T-cell populations rapidly increased to around 7,600 cells/mm³ by day 56 before declining and stabilizing at nearly 1,000 cells/mm³ through day 350 (**Figure 5B**). Activated cytotoxic T cells reached a peak of nearly 1,000 cells/mm³ by day 50 and remained elevated during the vaccination period, followed by a gradual decline accompanied by an increase in resting Tc cell populations (**Figure 5C**). In addition, active B-lymphocyte populations increased to around 750 cells/mm³ around day 56 and subsequently stabilized at 480–500 cells/mm³ during the later stages of the simulation (**Figure 5D**).

**Figure 5:**
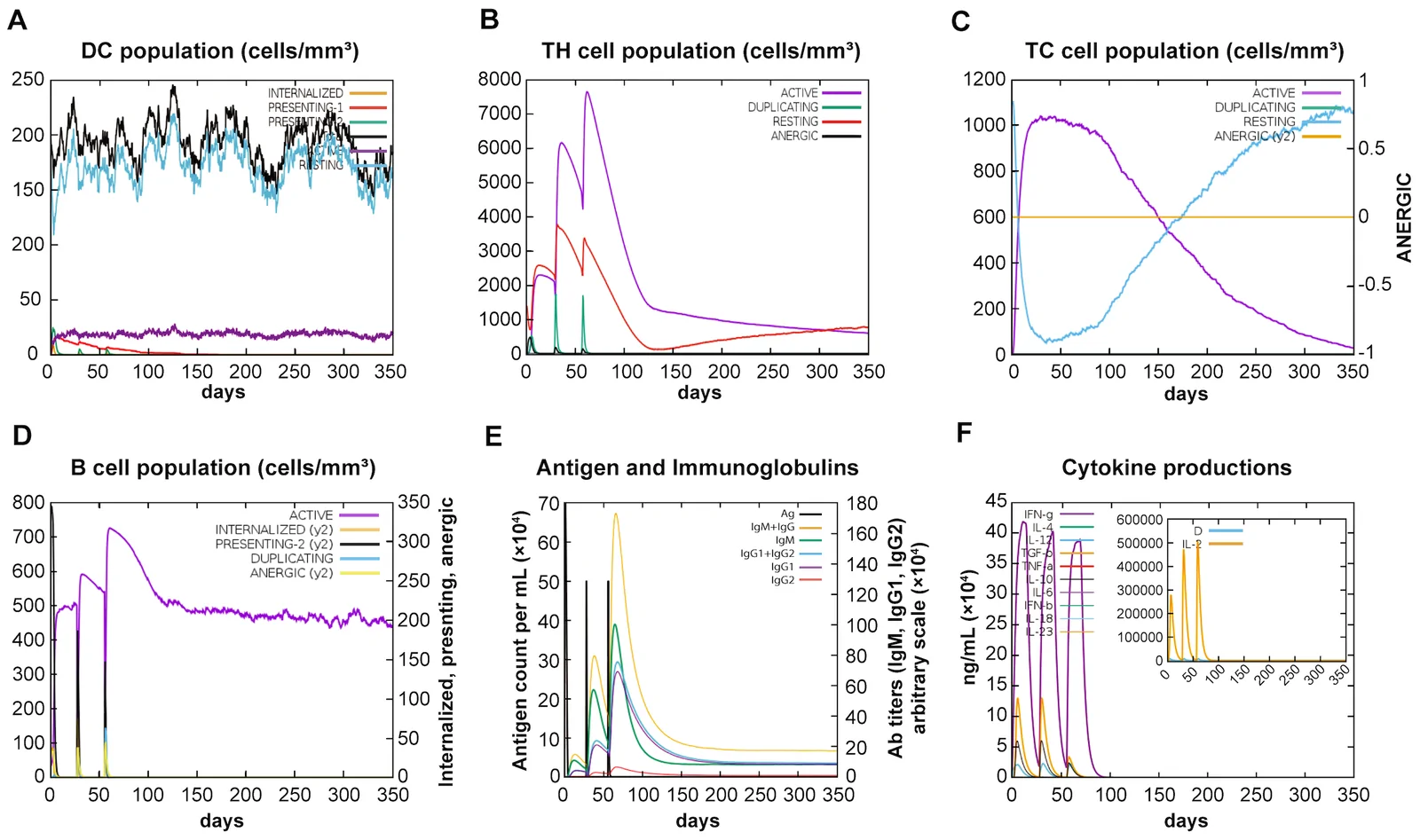
*In silico* immune simulation of the DENVAC/4 vaccine candidate using the C-ImmSim platform over a 350-day simulation period. Population dynamics of **(A)** dendritic cells (DCs), **(B)** T helper (TH) cells, **(C)** cytotoxic T cells (TCs), and **(D)** B cells following vaccine administration. **(E)** Antigen and immunoglobulin response profiles over time, including IgM, IgG1, and IgG2. **(F)** Cytokine profile.

Antigen and immunoglobulin profiling further demonstrated rapid humoral immune activation following DENVAC/4 administration (**Figure 5E**). Antigen levels increased transiently following each vaccine administration before declining. IgM responses increased markedly after booster administration, while combined IgM+IgG antibody titers reached a peak of nearly 170,000 by day 60 before gradually declining during the later stages of the simulation. IgG1 and combined IgG1+IgG2 responses also increased substantially following booster administration and remained detectable throughout the 350-day observation period, whereas IgG2 responses remained comparatively low. Cytokine profiling demonstrated transient increases following each vaccine administration, with IFN-γ showing the strongest response and reaching around 420,000 ng/mL during the early and booster phases (**Figure 5F**). IL-2 production was also markedly increased, with peak levels exceeding 500,000 ng/mL, while moderate increases in TGF-β, IL-10, and IL-12 were observed during the early immune response. Most cytokine responses peaked during the vaccination period and subsequently declined toward baseline levels during the later stages of the simulation.

### 3.8 HLA population coverage analysis

Population coverage analysis demonstrated broad global HLA recognition of the predicted epitopes. At the worldwide level, the projected population coverage was 68.55% for HLA class I epitopes, 93.79% for HLA class II epitopes, and 98.05% for the combined classes (**Supplementary File S10**). For HLA class I epitopes, the highest coverage was observed in Europe (80.31%), North America (70.43%), Taiwan (68.65%), and the Philippines, whereas lower coverage values were found in Central America, South Africa, Indonesia, and South Asia (**Figures 6A, C**). For HLA class II epitopes, the highest coverage was identified in North America (99.87%), Europe (99.58%), South Asia (97.70%), and Central Africa (95.12%), while lower coverage was observed in the Philippines, Central America, Borneo, and Vietnam (**Figures 6B, C**). The combined population coverage reached 99.96% in North America, 99.92% in Europe, 98.45% in South Asia, and 96.87% in Central Africa, whereas lower combined coverage values were detected in Central America, Borneo, South Africa, and Vietnam.

**Figure 6:**
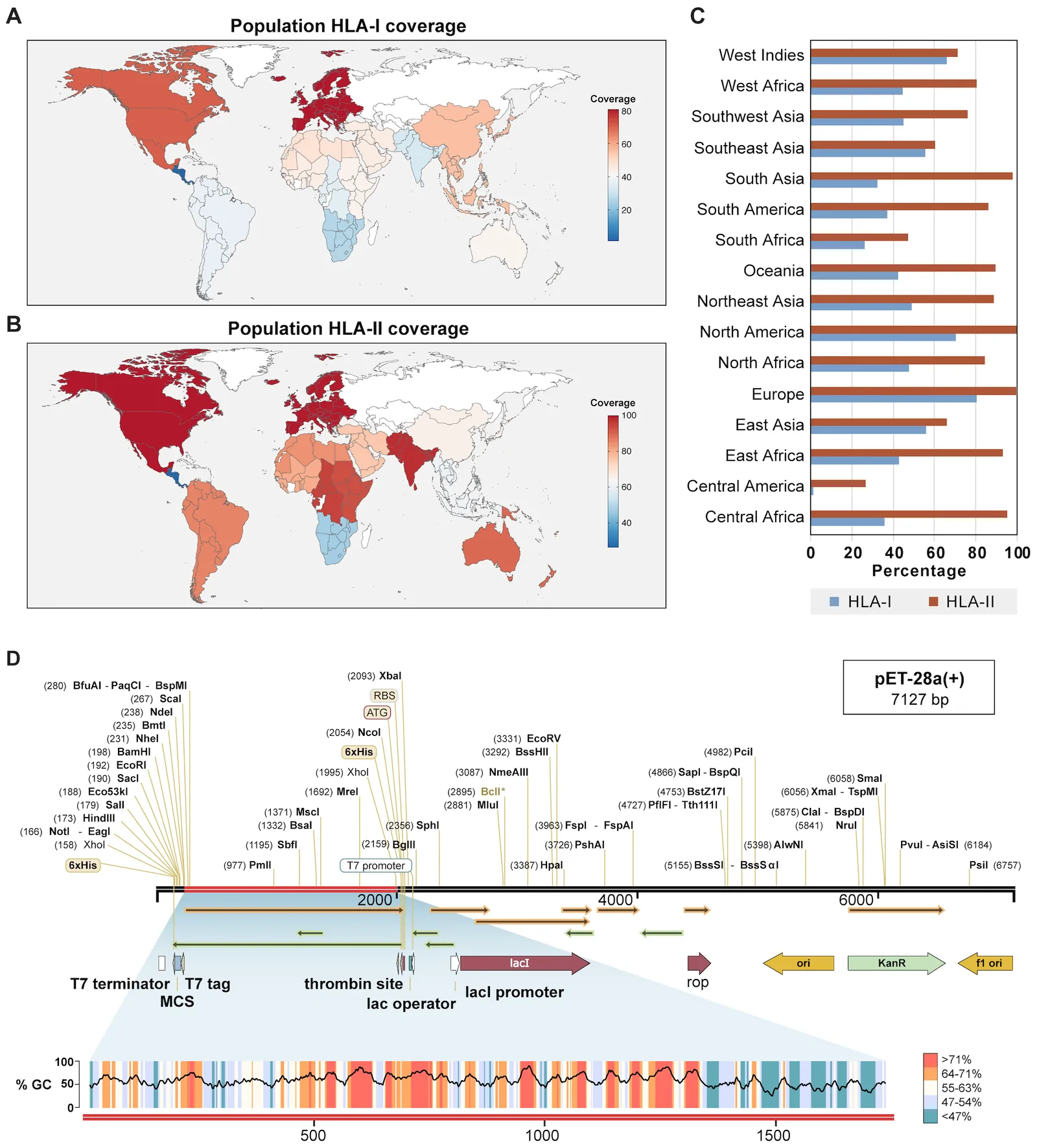
Global population coverage analysis and recombinant expression vector design for the DENVAC/4 vaccine candidate. World maps showing **(A)** predicted HLA class I and **(B)** HLA class II population coverage of the selected T-cell epitopes across different geographic regions. **(C)** Predicted population coverage for HLA class I and II epitopes across 16 global regions. **(D)** Linear plasmid map of the recombinant pET-28a(+) expression vector carrying the DENVAC/4 construct, annotated with major functional elements and restriction enzyme sites.

### 3.9 Codon optimization and *in silico* cloning of the multi-epitope vaccine construct

The designed multi-epitope vaccine protein sequence was back-translated into a DNA sequence of 1752 bp using EMBOSS Backtranseq. The resulting nucleotide sequence was subsequently optimized for expression in *Escherichia coli* using the codon optimization tool provided by VectorBuilder Codon Optimization Tool (https://en.vectorbuilder.com/tool/codon-optimization.html). Following optimization, the sequence exhibited a codon adaptation index (CAI) of 0.91 and a GC content of 59.42%, both of which fall within the optimal range for efficient gene expression in *E. coli* (**Supplementary File S11**). These results indicate a high potential for effective transcription and translation in the selected host system.

To enable recombinant expression, restriction sites for NdeI and XhoI were incorporated at the 5′ and 3′ ends of the optimized gene, respectively. The optimized gene was then successfully cloned *in silico* into the pET-28a(+) expression vector under the control of the T7 promoter (**Figure 6D**). The construct also included a 6×His-tag to facilitate downstream purification. The final recombinant plasmid construct was verified using SnapGene, confirming correct insertion, orientation, and maintenance of the proper reading frame.

## 4 Discussion

The development of an effective dengue vaccine remains particularly challenging because protective immunity must be achieved against all four DENV serotypes without inducing imbalanced or potentially pathogenic immune responses. In this context, the use of selective epitope-based vaccine design may offer advantages over conventional whole-protein approaches by limiting unnecessary antigenic regions that could contribute to non-protective or potentially dysregulated immune responses (Li et al., 2025; Parvizpour et al., 2020). The design strategy employed in DENVAC/4, which utilizes the non-structural proteins NS3 and NS5 for the selection of MHC class I and II epitopes while using the envelope (E) protein for B-cell epitope prediction, represents a rational and immunologically balanced approach to dengue vaccine development. This strategy is supported by increasing evidence demonstrating that optimal protective immunity against dengue virus requires coordinated activation of both cellular and humoral immune responses. NS3 and NS5 are among the most conserved proteins across DENV serotypes, reflecting functional constraints imposed by their essential roles in viral replication, polyprotein processing, and RNA synthesis. These constraints limit the accumulation of mutations and contribute to the preservation of conserved amino acid regions across serotypes (Khan et al., 2008; Nannetti et al., 2025).

However, although NS3 and NS5 proteins contain highly conserved regions among dengue serotypes, these sequences could not be universally utilized for epitope selection because effective vaccine design also requires compatibility with both HLA class I and class II molecules (Ahmed et al., 2020). In the present study, epitope screening was conducted using stringent immunogenetic criteria, including the exclusion of alleles previously associated with increased susceptibility to dengue infection or severe disease outcomes. HLA-A*03 and HLA-A*24 have been associated with an elevated risk of dengue hemorrhagic fever, particularly in Southeast Asian populations, whereas HLA-A*33 and HLA-B*44 appear to confer protective effects against severe dengue manifestations (de Alencar et al., 2013; Ghosh et al., 2025; Nguyen et al., 2008). Among the selected epitopes, only three CTL epitopes, MYFHRRDLR, YFHRRDLRL, and KGSRAIWYM demonstrated complete conservancy across all four DENV serotypes (100%), while several additional CTL and HTL epitopes retained moderate conservancy levels ranging from 50-75%, supporting broader serotype inclusivity. Meanwhile, many HTL epitopes were conserved in only a single serotype (25%), indicating that epitope selection was not restricted to universally conserved regions alone, but also considered overall immunogenic potential across the tetravalent construct. This approach may help maintain broader HLA-restricted immune recognition while accounting for the substantial genetic diversity of dengue viruses (Grifoni et al., 2017). There are many recent studies exploring tetravalent dengue multi-epitope vaccine design, with epitope conservation and HLA population coverage commonly considered during candidate selection. Saha et al. (2024) and Ferdousy et al. (2026) reported 99.70% and 97.35% worldwide combined population coverage, respectively. DENVAC/4 achieved a comparable combined coverage of 98.05%, with HLA class II coverage of 93.79%, exceeding the 81.81% reported in both studies, although its HLA class I coverage was lower than that of Saha et al. (98.55%) and Ferdousy et al. (85.42%) (Ferdousy et al., 2026; Saha et al., 2024). These findings indicate that incorporating dengue-associated HLA profiles into the selection strategy did not substantially reduce the overall predicted population coverage of DENVAC/4, which remained comparable to that of recent tetravalent multi-epitope dengue vaccine candidates. Although conserved regions were not specifically prioritized during T-cell epitope selection, the selected epitopes nevertheless spanned all four DENV serotypes, indicating that the selection strategy captured cross-serotype T-cell targets without relying primarily on sequence conservation.

The envelope (E) protein, particularly domain III (EDIII), is widely recognized as the principal target of neutralizing antibodies (Williams et al., 2012). The E protein mediates viral attachment and membrane fusion during host-cell entry and contains immunodominant antigenic regions accessible on the virion surface (Fibriansah et al., 2015; Zhang et al., 2017). B-cell epitope design was specifically focused on EDIII, with the three-dimensional structure first examined to identify surface-exposed regions that are accessible to antibody binding. These structurally accessible regions were then subjected to linear B-cell epitope prediction and screened for favorable antigenic properties, thereby prioritizing epitopes with greater potential for antibody recognition. This structure-guided strategy was intended to preferentially retain epitopes associated with neutralizing antibody responses while minimizing the inclusion of broadly cross-reactive regions commonly implicated in antibody-dependent enhancement (ADE). Notably, DENV-1, DENV-2, and DENV-4 each contained two predicted high-scoring epitopes, whereas DENV-3 exhibited a more limited epitope distribution with only a single suitable EDIII-derived candidate identified. Most of these candidate epitopes were localized within the FG loop or the DIII-DI hinge region of EDIII, both of which are structurally exposed regions implicated in antibody recognition and viral receptor interaction. Previous studies have shown that epitopes within these regions are frequently associated with serotype-specific neutralizing antibody responses rather than broadly cross-reactive antibodies linked to ADE (Sevvana and Kuhn, 2020). Importantly, several of the predicted EDIII epitopes demonstrated close correspondence with experimentally reported antibody-reactive sequences across all four DENV serotypes. In particular, the DENV-1 epitope YIVVGAGEKALKQCWF (DENV-1), the DENV-2 epitopes DSYIIIGVEPGQLKLN and KGMSYSMCTGKFKVVK (DENV-2), the DENV-3 epitope KGMSYAMCLNTFVLKK (DENV-3), and the DENV-4 epitope PPFGDSYIVIGVGNSA (DENV-4) showed sequence similarity to previously reported epitopes in the IEDB database. These correspondences provide independent support for the immunological relevance of the structure-guided epitope selection and suggest that the approach was able to identify antibody-recognized regions without relying solely on previously characterized epitope sequences.

DENVAC/4 consisted of 584 amino acids and incorporated multiple epitope regions and functional components while maintaining favorable predicted stability, as supported by the low instability index. This demonstrates that the incorporation of multiple antigenic components did not substantially compromise the overall physicochemical properties of the construct, which is a critical consideration for multi-epitope vaccine design (Alizadeh et al., 2022; Naveed et al., 2021). The predicted globular conformation suggests that the construct may adopt an organized three-dimensional architecture, which is relevant because protein folding and structural stability can influence the immunogenic properties of protein-based vaccine antigens (Scheiblhofer et al., 2017). Furthermore, the absence of predicted transmembrane helices indicates that DENVAC/4 does not possess apparent membrane-spanning regions, which may support its potential expression as a soluble recombinant protein by reducing the likelihood of membrane insertion during protein production.

The incorporation of β-defensin as an intrinsic adjuvant provides an additional immunological rationale for the DENVAC/4 design. Beyond its antimicrobial activity, β-defensin possesses immunomodulatory properties, including the ability to chemoattract immature dendritic cells and promote dendritic-cell activation and maturation through chemokine receptor- and Toll-like receptor-mediated mechanisms (Hoover et al., 2003). These properties may facilitate antigen uptake and presentation, thereby supporting the development of antigen-specific adaptive responses. Consistent with this immunological rationale, the *in silico* immune simulation of DENVAC/4 predicted the induction of both humoral and cellular immune responses, characterized by increased T-helper and T-cytotoxic cell populations, IFN-γ production, and IgG1 and IgG2 responses. This predicted immune profile aligns with the immunomodulatory effects previously described for β-defensins, particularly their capacity to promote dendritic-cell activation and type 1-polarized adaptive immune responses (Biragyn et al., 2002). In addition, the predicted interactions of DENVAC/4 with TLR2 and TLR4/MD2 may provide a mechanistic basis for its immunostimulatory potential. The more favorable interaction predicted for TLR4/MD2 suggests that this receptor may represent a particularly relevant pathway for recognition of the vaccine construct. However, computational binding analyses alone cannot establish receptor activation or downstream signaling. Additionally, although the TLR2–DENVAC/4 complex exhibited a higher number of hydrogen bonds, these interactions alone may not be sufficient to determine the overall binding strength. Other intermolecular interactions may also contribute to the predicted binding affinity. Importantly, the exceptionally low PRODIGY-predicted Kd (8.6×10^−20^ M) should be interpreted cautiously as a model-derived estimate rather than an experimentally determined binding affinity. The highly negative docking energy (-26 kcal/mol) and the absence of entropic contributions in the MM-GBSA calculation may result in overestimation of the absolute binding strength. Thus, these computational results should be interpreted as supporting the predicted receptor–vaccine interaction rather than quantitative evidence of exceptionally high-affinity binding. Engagement of TLR2 and TLR4 has been associated with dendritic cell maturation, macrophage activation, cytokine production, and enhanced antigen presentation, which may support the development of adaptive immune responses (Mukherjee et al., 2016; Zanin-Zhorov and Cohen, 2013). In this context, The interaction of DENVAC/4 with these receptors may contribute to the innate immune environment that supports subsequent activation of T-helper and cytotoxic T cells.

The molecular dynamics simulations further supported the docking results by demonstrating that DENVAC/4 maintained stable interactions with both TLR2 and TLR4/MD2 during the simulations. The RMSF analysis showed greater residue-level fluctuations in the vaccine component, predominantly at terminal and loop-exposed regions. These findings suggest that these regions retain greater conformational flexibility during receptor binding. Within DENVAC/4, higher fluctuations were mainly observed in the N-terminal adjuvant, CTL epitope, and C-terminal B-cell epitope regions. In contrast, the HTL epitope region exhibited comparatively fewer pronounced fluctuations. The regions showing greater flexibility also contributed to the docking interfaces. Interacting residues were identified within the adjuvant and CTL epitope regions, with additional contacts in the B-cell epitope region. This pattern suggests that local flexibility within these vaccine regions may facilitate conformational adjustment at receptor-contacting sites, supporting the accommodation of DENVAC/4 upon binding to TLR2 and TLR4/MD2 without compromising overall structural integrity. This interpretation was supported by the stable RMSD, SASA, and radius of gyration profiles, persistent hydrogen-bond interactions, and MM/GBSA profiles. These results indicated that local fluctuations did not cause substantial global structural disruption and supported the interaction of DENVAC/4 with both receptors, particularly TLR4/MD2.

Furthermore, the codon optimization and *in silico* cloning results suggest that DENVAC/4 may be efficiently expressed in *Escherichia coli* using the pET-28a(+) expression system. The high codon adaptation index and suitable GC content indicate compatibility with the bacterial translational machinery, supporting the potential for improved recombinant protein production (Zaytsev et al., 2024). The predicted solubility of DENVAC/4 also suggested a suitable profile for recombinant expression in *E. coli*. The incorporation of a 6×His-tag may improve the practicality of downstream vaccine development by simplifying recombinant protein purification and facilitating subsequent biochemical and structural characterization of the expressed construct.

Despite the promising findings, several limitations should be acknowledged. The selected epitopes and predicted immune responses remain computational predictions and may not fully reflect *in vivo* immunogenicity or antigen processing. Although epitopes covering all four DENV serotypes were included in this study, sequence conservation was assessed using only one representative sequence for each serotype. Given the substantial genetic diversity among DENV strains, including variation within individual serotypes, this approach may not fully capture the broader sequence diversity and may affect the predicted coverage and conservation of the selected epitopes. In addition, molecular docking and immune simulation analyses represent simplified models that cannot completely capture the complexity of host immune responses. The molecular dynamics (MD) simulations and C-ImmSim analysis were performed as single runs without replicate simulations. Given the potential for frame-to-frame correlation in MD-based analyses and the stochastic nature of immune simulations, the observed trends should therefore be interpreted with caution, and replicate simulations would be beneficial to further assess the robustness and reproducibility of these findings. Therefore, experimental validation is required to confirm the immunogenicity, stability, antigenicity, and protective efficacy of DENVAC/4 through appropriate *in vitro* and *in vivo* studies.

## 5 Conclusion

This study presents a comprehensive immunoinformatic-guided framework for the development of DENVAC/4 as a rationally designed multi-epitope tetravalent dengue vaccine candidate. The incorporation of selected immunogenic epitopes together with intrinsic innate immune stimulatory components resulted in a construct with favorable predicted structural characteristics, broad predicted population coverage, stable receptor interactions, and simulated immune responses. By integrating NS3/NS5-derived T-cell epitopes with E protein-derived B-cell epitopes, DENVAC/4 was designed to target both cellular and humoral immune responses across all four dengue virus serotypes. This strategy may provide an alternative approach to conventional E protein-focused vaccine platforms by incorporating both T-cell and B-cell epitopes while considering the potential risk of antibody-dependent enhancement. Collectively, these findings provide computational support for the biological plausibility of DENVAC/4 and its potential as a candidate for further experimental investigation. Nevertheless, these findings are derived from *in silico* analyses and warrant further validation through appropriate *in vitro* and *in vivo* studies in future research to assess the immunogenicity, safety, and protective efficacy of DENVAC/4.

## Data Availability

The three-dimensional structures of TLR2 (PDB ID: 2Z7X; https://www.rcsb.org/structure/2Z7X) and TLR4/MD2 (PDB ID: 4G8A; https://www.rcsb.org/structure/4G8A) were retrieved from the RCSB Protein Data Bank. All datasets generated during this study, including protein sequences and 3D structural models, have been deposited in Zenodo and are publicly accessible at https://doi.org/10.5281/zenodo.22661207.
