# Supplementary material for: Design of a tetravalent dengue vaccine incorporating T-cell epitopes from NS3 and NS5 proteins and B-cell epitopes from the envelope protein using an immunoinformatics approach
Source: Front Cell Infect Microbiol. 2026 Oct 7;16:1934834. doi: 10.3389/fcimb.2026.1934834 (PMC13683149; doi:10.3389/fcimb.2026.1934834)
Supplement: Supplementary file 1 [file DataSheet1.pdf]

DENVAC/4 sequence

[Bete-defensine-AAY-CTL epitopes-GPGPG-HTL epitopes-KK-LBL epitopes]

GIINTLQKYYCRVRGGRCAVLSCLPKEEQIGKCSTRGRKCCRRKKEAAKMYFHRRDLRAAYYFH
RRDLRLAAYRPRWLDARVAAYWLGARFLEFAAYKGSRAIWYMAAYLEFEALGFLAAYEEALRGL
PIAAYCHATFTMRLAAYFVGKTVWFVAAYVEMGEAAAIAAYAHFTDPASIGPGPGRGYISTRVE
MGEAAAGPGPGRVGMGEAAAIFMTATGPGPGGTYVSAIAQAKASQEGPGPGWHVTRGAVLMYQG
KRGPGPGKTWAYHGSYEVKPSGGPGPGDFVVTTDISEMGANFGPGPGNPYMPSVIEKMEALQGP
GPGSAAQRRGRIGRNPKNGPGPGAMYADDTAGWDTRITGPGPGWLETKGVERLKRMAIGPGPGR
SGILEVDRTEAKSAGPGPGWFSRENSLSGVEGEGGPGPGWLGARFLEFEALGFLGPGPGWALLG
KKKNPRLCTRKKPPFGDSYIVIGVGNSAKKYIVVGAGEKALKQCWFKKDSYIIIGVEPGQLKLN
KKKGMSYVMCTGSFKLEKKKKGMSYSMCTGKFKVVKKKKGMSYAMCLNTFVLKKKKKGMSYTMC
SGKFSIDK
