# Supplementary material for: Design of a tetravalent dengue vaccine incorporating T-cell epitopes from NS3 and NS5 proteins and B-cell epitopes from the envelope protein using an immunoinformatics approach
Source: Front Cell Infect Microbiol. 2026 Oct 7;16:1934834. doi: 10.3389/fcimb.2026.1934834 (PMC13683149; doi:10.3389/fcimb.2026.1934834)
Supplement: Supplementary file 2 [file DataSheet2.pdf]

Back-translated DNA sequence (EMBOSS Backtranseq)

GGCATCATCAACACCCTGCAGAAGTACTACTGCAGGGTGAGGGGCGGCAGGTGCGCCGTG
CTGAGCTGCCTGCCCAAGGAGGAGCAGATCGGCAAGTGCAGCACCAGGGGCAGGAAGTGC
TGCAGGAGGAAGAAGGAGGCCGCCAAGATGTACTTCCACAGGAGGGACCTGAGGGCCGCC
TACTACTTCCACAGGAGGGACCTGAGGCTGGCCGCCTACAGGCCCAGGTGGCTGGACGCC
AGGGTGGCCGCCTACTGGCTGGGCGCCAGGTTCCTGGAGTTCGCCGCCTACAAGGGCAGC
AGGGCCATCTGGTACATGGCCGCCTACCTGGAGTTCGAGGCCCTGGGCTTCCTGGCCGCC
TACGAGGAGGCCCTGAGGGGCCTGCCCATCGCCGCCTACTGCCACGCCACCTTCACCATG
AGGCTGGCCGCCTACTTCGTGGGCAAGACCGTGTGGTTCGTGGCCGCCTACGTGGAGATG
GGCGAGGCCGCCGCCATCGCCGCCTACGCCCACTTCACCGACCCCGCCAGCATCGGCCCC
GGCCCCGGCAGGGGCTACATCAGCACCAGGGTGGAGATGGGCGAGGCCGCCGCCGGCCCC
GGCCCCGGCAGGGTGGGCATGGGCGAGGCCGCCGCCATCTTCATGACCGCCACCGGCCCC
GGCCCCGGCGGCACCTACGTGAGCGCCATCGCCCAGGCCAAGGCCAGCCAGGAGGGCCCC
GGCCCCGGCTGGCACGTGACCAGGGGCGCCGTGCTGATGTACCAGGGCAAGAGGGGCCCC
GGCCCCGGCAAGACCTGGGCCTACCACGGCAGCTACGAGGTGAAGCCCAGCGGCGGCCCC
GGCCCCGGCGACTTCGTGGTGACCACCGACATCAGCGAGATGGGCGCCAACTTCGGCCCC
GGCCCCGGCAACCCCTACATGCCCAGCGTGATCGAGAAGATGGAGGCCCTGCAGGGCCCC
GGCCCCGGCAGCGCCGCCCAGAGGAGGGGCAGGATCGGCAGGAACCCCAAGAACGGCCCC
GGCCCCGGCGCCATGTACGCCGACGACACCGCCGGCTGGGACACCAGGATCACCGGCCCC
GGCCCCGGCTGGCTGGAGACCAAGGGCGTGGAGAGGCTGAAGAGGATGGCCATCGGCCCC
GGCCCCGGCAGGAGCGGCATCCTGGAGGTGGACAGGACCGAGGCCAAGAGCGCCGGCCCC
GGCCCCGGCTGGTTCAGCAGGGAGAACAGCCTGAGCGGCGTGGAGGGCGAGGGCGGCCCC
GGCCCCGGCTGGCTGGGCGCCAGGTTCCTGGAGTTCGAGGCCCTGGGCTTCCTGGGCCCC
GGCCCCGGCTGGGCCCTGCTGGGCAAGAAGAAGAACCCCAGGCTGTGCACCAGGAAGAAG
CCCCCCTTCGGCGACAGCTACATCGTGATCGGCGTGGGCAACAGCGCCAAGAAGTACATC
GTGGTGGGCGCCGGCGAGAAGGCCCTGAAGCAGTGCTGGTTCAAGAAGGACAGCTACATC
ATCATCGGCGTGGAGCCCGGCCAGCTGAAGCTGAACAAGAAGAAGGGCATGAGCTACGTG
ATGTGCACCGGCAGCTTCAAGCTGGAGAAGAAGAAGAAGGGCATGAGCTACAGCATGTGC
ACCGGCAAGTTCAAGGTGGTGAAGAAGAAGAAGGGCATGAGCTACGCCATGTGCCTGAAC
ACCTTCGTGCTGAAGAAGAAGAAGAAGGGCATGAGCTACACCATGTGCAGCGGCAAGTTC
AGCATCGACAAG

Optimized DNA DENVAC4
GC=59.42%, CAI=0.91

GGCATCATCAACACCCTGCAGAAGTACTATTGCCGAGTGAGGGGGGGCAGATGTGCCGTG
CTGAGCTGTCTGCCCAAGGAGGAGCAGATTGGCAAGTGTTCCACTCGCGGCCGGAAGTGC
TGCAGGAGAAAGAAGGAAGCCGCCAAGATGTACTTTCATCGGAGAGATCTGAGGGCCGCA
TACTACTTCCACCGCAGAGACCTGAGACTGGCTGCCTACAGGCCTCGCTGGCTGGACGCC
AGGGTCGCAGCCTATTGGCTGGGTGCCAGATTTCTGGAATTTGCCGCCTATAAGGGAAGT
AGGGCCATTTGGTATATGGCCGCCTATCTGGAGTTTGAGGCACTGGGCTTTCTGGCAGCC
TATGAGGAGGCACTGAGGGGACTGCCAATTGCTGCCTACTGTCATGCCACTTTTACCATG
AGGCTGGCCGCCTACTTCGTGGGCAAAACCGTGTGGTTCGTGGCTGCCTACGTGGAGATG
GGCGAAGCCGCTGCGATCGCCGCCTATGCACACTTCACCGACCCCGCTTCCATCGGCCCT
GGCCCTGGCAGGGGATACATTAGCACCAGGGTGGAGATGGGCGAAGCGGCTGCCGGCCCT
GGCCCAGGCCGGGTGGGAATGGGCGAGGCAGCCGCCATTTTTATGACAGCCACAGGCCCT
GGGCCTGGAGGGACCTACGTCAGTGCCATCGCCCAGGCTAAGGCCAGTCAGGAGGGCCCT
GGCCCTGGCTGGCACGTGACCCGGGGCGCCGTGCTGATGTACCAGGGCAAACGGGGCCCA
GGACCTGGCAAGACTTGGGCCTATCATGGGTCCTATGAGGTGAAACCCTCCGGAGGTCCC
GGTCCAGGAGACTTCGTGGTGACAACCGACATTAGCGAAATGGGAGCCAACTTTGGACCT
GGCCCCGGCAATCCCTACATGCCCAGCGTGATCGAAAAGATGGAGGCCCTGCAGGGCCCC
GGCCCAGGCAGCGCCGCCCAGAGAAGAGGAAGAATCGGAAGGAACCCAAAGAATGGCCCA
GGCCCTGGCGCTATGTACGCCGATGATACTGCCGGCTGGGACACTCGCATCACAGGGCCC
GGCCCAGGGTGGCTCGAAACAAAAGGCGTGGAGAGGCTGAAAAGAATGGCCATCGGGCCG
GGTCCAGGCCGCAGTGGCATCCTGGAGGTGGATAGAACCGAGGCAAAGTCCGCCGGCCCT
GGACCTGGCTGGTTCAGCAGGGAGAACTCCCTGTCCGGCGTCGAGGGAGAGGGCGGCCCA
GGCCCCGGGTGGCTGGGGGCCAGATTTCTGGAGTTCGAGGCTCTGGGGTTCCTGGCCCCC
GGCCCAGGATGGGCCCTGCTGGGTAAGAAGAAGAATCCCAGACTGTGCACAAGAAAAAAG
CCACCCTTCGGGGATTCCTATATCGTGATCGGCGTGGGCAATAGCGCCAAAAAGTATATC
GTGGTGGGAGCTGGGGAGAAGGCCCTGAAACAGTGTTGGTTTAAAAAGGACTCTTACATT
ATCATTGGGGTGGAACCAGGGCAGCTGAAGCTGAATAAGAAGAAGGGCATGTCTTACGTC
ATGTGCACAGGAAGCTTCAAGCTGGAGAAAAAAAAGAAGGGCATGAGCTATTCTATGTGC
ACCGGGAAGTTCAAGGTGGTGAAAAAGAAGAAGGGCATGAGCTACGCCATGTGCCTGAAC
ACCTTCGTGCTGAAAAAAAAGAAGAAGGGAATGAGCTATACCATGTGCAGCGGCAAGTTC
AGCATCGATAAG
