# Supplementary material for: Design of a tetravalent dengue vaccine incorporating T-cell epitopes from NS3 and NS5 proteins and B-cell epitopes from the envelope protein using an immunoinformatics approach
Source: Front Cell Infect Microbiol. 2026 Oct 7;16:1934834. doi: 10.3389/fcimb.2026.1934834 (PMC13683149; doi:10.3389/fcimb.2026.1934834)
Supplement: Supplementary file 3 [file Table2.docx]

```

CLUSTAL O(1.2.4) multiple sequence alignment

E-protein

Abbreviation
E1: E-protein Dengue 1
E2: E-protein Dengue 2
E3: E-protein Dengue 3
E4: E-protein Dengue 4

E4      MRCVGVGNRDFVEGVSGGAWVDLVLEHGGCVTTMAQGKPTLDFELTKTTAKEVALLRTYC	60
E2      MRCIGISNRDFVEGVSGGSWVDIVLEHGSCVTTMAKNKPTLDFELIETEAKQPATLRKYC	60
E1      MRCVGIGSRDFVEGLSGATWVDVVLEHGSCVTTMAKDKPTLDIELLKTEVTNPAVLRKLC	60
E3      MRCVGVGNRDFVEGLSGATWVDVVLEHGGCVTTMAKNKPTLDIELQKTEATQLATLRKLC	60
        ***:*:..******:**.:***:*****.******:.*****:** :* ..: * **. *

E4      IEASISNITTATRCPTQGEPYLKEEQDQQYICRRDVVDRGWGNGCGLFGKGGVVTCAKFS	120
E2      IEAKLTNTTTDSRCPTQGEPSLNEEQDKRFVCKHSMVDRGWGNGCGLFGKGGIVTCAMFT	120
E1      IEAKISNTTTDSRCPTQGEATLVEEQDANFVCRRTFVDRGWGNGCGLFGKGSLLTCAKFK	120
E3      IEGKITNITTDSRCPTQGEAILPEEQDQNYVCKHTYVDRGWGNGCGLFGKGSLVTCAKFQ	120
        **..::* ** :*******  * **** .::*::  ***************.::*** *

E4      CSGKITGNLVQIENLEYTVVVTVHNGDTHAVGNDTSNHGVTAMITPRSPSVEVKLPDYGE	180
E2      CKKNMKGKVVQPENLEYTIVITPHSGEEHAVGNDTGKHGKEIKITPQSSITEAELTGYGT	180
E1      CVTKLEGKIVQYENLKYSVIVTVHTGDQHQVGNETTEHGTIATITPQAPTSEIQLTDYGA	180
E3      CLESIEGKVVQHENLKYTVIITVHTGDQHQVGNETQ--GVTAEITSQASTAEAILPEYGT	178
        *  .: *::** ***:*::::* *.*: * ***:*   *    ** ::   *  *  **

E4      LTLDCEPRSGIDFNEMILMKMKKKTWLVHKQWFLDLPLPWTAGADTSEVHWNYKERMVTF	240
E2      VTMECSPRTGLDFNEMVLLQMENKAWLVHRQWFLDLPLPWLPGADTQGSNWIQKETLVTF	240
E1      LTLDCSPRTGLDFNEMVLLTMKEKSWLVHKQWFLDLPLPWTSGASTSQETWNRQDLLVTF	240
E3      LGLECSPRTGLDFNEMILLTMKNKAWMVHRQWFFDLPLPWTSGATTKTPTWNRKELLVTF	238
        : ::*.**:*:*****:*: *::*:*:**:***:******  ** *.   *  :: :***

E4      KVPHAKRQDVTVLGSQEGAMHSALAGATEVDSGDGNHMFAGHLKCKVRMEKLRIKGMSYT	300
E2      KNPHAKKQDVVVLGSQEGAMHTALTGATEIQMSSGNLLFTGHLKCRLRMDKLQLKGMSYS	300
E1      KTAHAKKQEVVVLGSQEGAMHTALTGATEIQTSGTTTIFAGHLKCRLKMDKLTLKGMSYV	300
E3      KNAHAKKQEVVVLGSQEGAMHTALTGATEIQTSGGTSIFAGHLKCRLKMDKLKLKGMSYA	298
        *  ***:*:*.**********:**:****:: .. . :*:*****:::*:** :*****

E4      MCSGKFSIDKEMAETQHGTTVVKVKYEGAGAPCKVPIEIRDVNKEKVVGRIISSTPLAEN	360
E2      MCTGKFKVVKEIAETQHGTIVIRVQYEGDGSPCKIPFEIMDLEKRHVLGRLITVNPIVTE	360
E1      MCTGSFKLEKEVAETQHGTVLVQVKYEGTDAPCKIPFSTQDEKGVT-QNRLITANPIVTD	359
E3      MCLNTFVLKKEVSETQHGTILIKVEYKGEDAPCKIPFSTEDGQGKAHNGRLITANPVVTK	358
        ** ..* : **::****** :::*:*:* .:***:*:.  * :     .*:*: .*:. .

E4      TNSVTNIELEPPFGDSYIVIGVGNSALTLHWFRKGSSIGKMFESTYRGAKRMAILGETAW	420
E2      KDSPVNIEAEPPFGDSYIIIGVEPGQLKLNWFKKGSSIGQMIETTMRGAKRMAILGDTAW	420
E1      KEKPVNIETEPPFGESYIVVGAGEKALKQCWFKKGSSIGKMFEATARGARRMAILGDTAW	419
E3      KEEPVNIEAEPPFGESNIVIGIGDKALKINWYRKGSSIGKMFEATARGARRMAILGDTAW	418
        .:. .*** *****:* *::*     *.  *::******:*:*:* ***:******:***

E4      DFGSVGGLFTSLGKAVHQVFGSVYTTMFGGVSWMIRILIGFLVLWIGTNSRNTSMAMTCI	480
E2      DFGSLGGVFTSIGKALHQVFGAIYGAAFSGVSWIMKILIGVIITWIGMNSRSTSLSVSLV	480
E1      DFGSIGGVFTSVGKLVHQVFGTAYGVLFSGVSWTMKIGIGILLTWLGLNSRSTSLSMTCI	479
E3      DFGSVGGVLNSLGKMVHQIFGSAYTALFSGVSWIMKIGIGVLLTWIGLNSKNTSMSFSCI	478
        ****:**::.*:** :**:**: * . *.**** ::* **.:: *:* **:.**::.: :

E4      AVGGITLFLGFTVQA	495
E2      LVGVVTLYLGVMVQA	495
E1      AVGMVTLYLGVMVQA	494
E3      AIGIITLYLGVVVQA	493
         :* :**:**. ***

____________________________________________________

NS3

Abbreviation
NS3/1: NS3 of Dengue virus serotypes 1
NS3/2: NS3 of Dengue virus serotypes 2
NS3/3: NS3 of Dengue virus serotypes 3
NS3/4: NS3 of Dengue virus serotypes 4

NS3/2   AGVLWDVPSPPPVGKAELEDGAYRIKQKGILGYSQIGAGVYKEGTFHTMWHVTRGAVLMH	60
NS3/4   SGALWDVPSPAATKKAALSEGVYRIMQRGLFGKTQVGVGIHMEGVFHTMWHVTRGSVICH	60
NS3/1   SGVLWDTPSPPEVERAVLDDGIYRIMQRGLLGRSQVGVGVFQDGVFHTMWHVTRGAVLMY	60
NS3/3   SGVLWDVPSPPETQKAELEEGVYRIKQQGIFGKTQVGVGVQKEGVFHTMWHVTRGAVLTH	60
        :*.***.***  . :* *.:* *** *:*::* :*:*.*:  :*.**********:*: :

NS3/2   KGKRIEPSWADVKKDLISYGGGWKLEGEWKEGEEVQVLAL-PGKNPRAVQTKPGLFKTNA	119
NS3/4   ETGRLEPSWADVRNDMISYGGGWRLGDKWDKEEDVQVLAIEPGKNPKHVQTKPGLFKTLT	120
NS3/1   QGKRLEPSWASVKKDLISYGGGWRFQGSWNTGEEVQVIAVEPGKNPKNVQTAPGTFKTPE	120
NS3/3   NGKRLEPNWASVKKDLISYGGGWRLSAQWQKGEEVQVIAVEPGKNPKNFQTMPGIFQTTT	120
        :  *:**.**.*::*:*******::  .*.  *:***:*: *****: .** ** *:*

NS3/2   GTIGAVSLDFSPGTSGSPIIDKKGKVVGLYGNGVVTRSGAYVSAIAQTEKSIE-DNPEIE	178
NS3/4   GEIGAVTLDFKPGTSGSPIINRKGKVIGLYGNGVVTKSGDYVSAITQAERIGEPDYEVDE	180
NS3/1   GEVGAIALDFKPGTSGSPIVNREGKIVGLYGNGVVTTSGTYVSAIAQAKASQEGPLPEIE	180
NS3/3   GEIGAIALDFKPGTSGSPIINREGKVVGLYGNGVVTKNGGYVSGIAQTNAEPDGPTPELE	180
        * :**::***.********::::**::********* .* ***.*:*::   :      *

NS3/2   DDIFRKRKLTIMDLHPGAGKTKRYLPAIVREAIKRGLRTLILAPTRVVAAEMEEALRGLP	238
NS3/4   EDIFRKKRLTIMDLHPGAGKTKRILPSIVREALKRRLRTLILAPTRVVAAEMEEALRGLP	240
NS3/1   DEVFRKRNLTIMDLHPGSGKTRRYLPAIVREAIRRNVRTLILAPTRVVASEMAEALKGMP	240
NS3/3   EEMFKKRNLTIMDLHP-SGKTRKYLPAIVREAIKRRLRTLILAPTRVVAAEMEEAMKGLP	239
        :::*:*:.******** :***:: **:*****::* :************:** **::*:*

NS3/2   IRYQTPAIRAEHTGREIVDLMCHATFTMRLLSPVRVPNYNLIIMDEAHFTDPASIAARGY	298
NS3/4   IRYQTPAVKSEHTGREIVDLMCHATFTTRLLSSTRVPNYNLIVMDEAHFTDPSSVAARGY	300
NS3/1   IRYQTTAVKSEHTGKEIVDLMCHATFTMRLLSPVRVPNYNMIIMDEAHFTDPASIARRGY	300
NS3/3   IRYQTTATKSEHTGREIVDLMCHATFTMRLLSPVRVPNYNLIIMDEAHFTDPASIAARGY	299
        ***** * ::****:************ **** .******:*:*********:*:* ***

NS3/2   ISTRVEMGEAAGIFMTATPPGSRDPFPQSNAPIMDEEREIPERSWSSGHEWVTDKFGKTV	358
NS3/4   ISTRVEMGEAAAIFMTATPPGATDPFPQSNSPIEDIEREIPERSWNTGFDWITDYQGKTV	360
NS3/1   ISTRVGMGEAAAIFMTATPPGSVEAFPQSNAVIQDEERDIPERSWNSGYEWITDFPGKTV	360
NS3/3   ISTRVGMGEAAAIFMTATPPGTADAFPQSNAPIQDEERDIPERSWNSGNEWITDFVGKTV	359
        ***** *****.*********: : *****: * * **:******.:* :*:**  ****

NS3/2   WFVPSIKAGNDIAACLRKNGKKVIQLSRKTFDSEYVKTRTNDWDFVVTTDISEMGANFKA	418
NS3/4   WFVPSIKAGNDIANCLRKSGKKVIQLSRKTFDTEYPKTKLTDWDFVVTTDISEMGANFRA	420
NS3/1   WFVPSIKSGNDIANCLRKNGKRVIQLSRKTFDTEYQKTKNNDWDYVVTTDISEMGANFRA	420
NS3/3   WFVPSIKAGNVIANCLRKNGKKVIQLSRKTFDTEYQKTKLNDWDFVVTTDISEMGANFIA	419
        *******:** ** ****.**:**********:** **: .***:************* *

NS3/2   E-RVIDPRRCMKPVILTDGEERVILAGPMPVTHSSAAQRRGRIGRNPKNENDQYIYMG-P	476
NS3/4   G-RVIDPRRCLKPVILPDGPERVILAGPIPVTPASAAQRRGRIGRNPAQEDDQYVFSGDP	479
NS3/1   DRRVIDPRRCLKPVILKDGPERVILAGPMPVTVASAAQRRGRIGRNQNKEGDQYVYMGQP	480
NS3/3   D-RVIDPRRCLKPVILTDGPERVILAGPMPVTVASAAQRRGRVGRNPQKENDQYIFMGQP	478
          ********:***** ** ********:*** :********:***  :*.***:: * *

NS3/2   LENDEDCAHWKEAKMLLDNINTPEGIIPSMFEPEREKVDAIDGEYRLRGEARKTFVDLMR	536
NS3/4   LKNDEDHAHWTEAKMLLDNIYTPEGIIPTLFGPEREKTQAIDGEFRLRGEQRKTFVELMR	539
NS3/1   LNNDEDHAHWTEAKMLLDNINTPEGIIPALFEPEREKSAAIDGEYRLRGEARKTFVELMR	540
NS3/3   LNKDEDHAHWTEAKMLLDNINTPEGIIPALFEPEREKSAAIDGEYRLKGESRKTFVELMR	538
        *::*** ***.********* *******::* *****  *****:**:** *****:***

NS3/2   RGDLPVWLAYRVAAEGINYADRRWCFDGIKNNQILEENVEVEIWTKEGERKKLKPRWLDA	596
NS3/4   RGDLPVWLSYKVASAGISYKDREWCFTGERNNQILEENMEVEIWTREGEKKKLRPRWLDA	599
NS3/1   RGDLPVWLSYKVASEGFQYSDRRWCFDGERNNQVLEENMDVEMWTKEGERKKLRPRWLDA	600
NS3/3   RGDLPVWLAHKVASEGIKYTDRKWCFDGERNNQILEENMDVEIWTKEGEKKKLRPRWLDA	598
        ********:::**: *:.* **.*** * :***:****::**:**:***:***:******

NS3/2   KIYSDPLALKEFKEFAAGRK	616
NS3/4   RVYADPMALKDFKEFASGRK	619
NS3/1   RTYSDPLALREFKEFAAGRR	620
NS3/3   RTYSDPLALKEFKDFAAGRK	618
        : *:**:**::**:**:**:


____________________________________________________


NS5

Abbreviation
NS5/1: NS5 of Dengue virus serotypes 1
NS5/2: NS5 of Dengue virus serotypes 2
NS5/3: NS5 of Dengue virus serotypes 3
NS5/4: NS5 of Dengue virus serotypes 4


NS54    GTGTTGETLGEKWKRQLNSLDRKEFEEYKRSGILEVDRTEAKSALKDGSKIKHAVSRGSS	60
NS52    GTGNIGETLGEKWKSRLNALGKSEFQIYK-SGIQEVDRTLAKEGIKRGETDHHAVSRGSA	59
NS51    GTGAKGKHWERNGKDRLNQLSKSEFNTYK-RGIMEVDRSEAKEGLKRGETTKHAVSRGTA	59
NS53    GTGSQGETLGEKWKKKLNQLSRKEFDLYKKSGITEVDRTEAKEGLKRGEITHHAVSRGSA	60
        ***  *:   .: * :** *.:.**: **  ** ****: **..:* *.  :******::

NS54    KIRWIVERGMVKPKGKVVDLGCGRGGWSYYMATLKNVTEVKGYTKGGPGHEEPIPMATYG	120
NS52    KLRWFVERNMVTPEGKVVDLGCGRGGWSYYCGGLKNVREVKGLTKGGPGHEEPIPMSTYG	119
NS51    KLRWFVERNLVKPEGKVIDLGCGRGGWSYYCAGLKKVTEVKGYTKGGPGHEEPIPMATYG	119
NS53    KLQWFVERNMVIPEGRVIDLGCGRGGWSYYCAGLKKVTEVRGYTKGGPGHEEPVPMSTYG	120
        *::*:***.:* *:*:*:************ . **:* **:* **********:**:***

NS54    WNLVKLHSGVDVFYKPTEQVDTLLCDIGESSSNPPTIEEGRTLRVLKMVEPWLSSKPEFC	180
NS52    WNLVRLQSGVDVFFTPPEKCDTLLCDIGESSPN-PT-EAGRTLRVLNLVENWLNNNTQFC	177
NS51    WNLVKLYSGKDVFFTPPEKCDTLLCDIGESSPN-PTIEEGRTLRVLKMVEPWLRG-NQFC	177
NS53    WNIVKLMSGKDVFYLPPEKCDTLLCDIGESSPS-PTVEESRTIRVLKMVEPWLKN-NQFC	178
        **:*:* ** ***: * *: *********** . ** * .**:***::** ** .  :**

NS54    IKVLNPYMPTVIEELEKLQRKHGGNLVRCPLSRNSTHEMYWVSGASGNIVSSVNTTSKML	240
NS52    IKVLNPYMPSVIEKMEALQRKYGGALVRNPLSRNSTHEMYWLSNASGNIVSSVNMISRML	237
NS51    IKILNPYMPSVVETLEQMQRKHGGMLVRNPLSRNSTHEMYWVSCGTGNIVSAVNMTSRML	237
NS53    IKVLNPYMPTVIEHLERLQRKHGGMLVRNPLSRNSTHEMYWISNGTGNIVSSVNMVSRLL	238
        **:******:*:* :* :***:** *** ************:* .:*****:**  *::*

NS54    LNRFTTRHRKPTYEKDVDLGAGTRSVSTETEKPDMTIIGRRLQRLQEEHKETWHYDQENP	300
NS52    INRFTMRHKKATYEPDVDLGSGTRNIGIESEIPNLDIIGKRIEKIKQEHETSWHYDQDHP	297
NS51    LNRFTMAHRKPTYERDVDLGAGTRHVAVEPEVANLDIIGQRIENIKHEHKSTWHYDEDNP	297
NS53    LNRFTMTHRRPTIEKDVDLGAGTRHVNAEPETPNMDVIGERIKRIKEEHSSTWHYDDENP	298
        :****  *:: * * *****:*** :  * *  :: :**.*::.::.**. :****:::*

NS54    YRTWAYHGSYEAPSTGSASSMVNGVVKLLTKPWDVIPMVTQLAMTDTTPFGQQRVFKEKV	360
NS52    YKTWAYHGSYETKQTGSASSMGNGVVRLLTKPWDVVPMVTQMAMTDTTPFGQQRVFKEKV	357
NS51    YKTWAYHGSYEVKPSGSASSMVNGVVKLLTKPWDAIPMVTQIAMTDTTPFGQQRVFKEKV	357
NS53    YKTWAYHGSYEVKATGSASSMINGVVKLLTKPWDVVPMVTQMAMTDTTPFGQQRVFKEKV	358
        *:*********.  :****** ****:*******.:*****:******************

NS54    DTRTPQPKPGTRMVMTTTANWLWALLGKKKNPRLCTREEFISKVRSNAAIGAVFQEEQGW	420
NS52    DTRTQEPKEGTKKLMKITAEWLWKELGKKKTPRMCTREEFTRKVRSNAALGAIFTDENKW	417
NS51    DTRTPKAKRGTAQIMEVTARWLWGFLSRNKKPRICTREEFTRKVRSNAAIGAVFVDENQW	417
NS53    DTRTPRPMPGTRKVMEITAEWLWRTLGRNKRPRLCTREEFTKKVRTNAAMGAVFTEENQW	418
        **** .   **  :*  **.***  *.::* **:******  ***:***:**:* :*: *

NS54    TSASEAVNDSRFWELVDKERALHQEGKCESCVYNMMGKREKKLGEFGRAKGSRAIWYMWL	480
NS52    KSAREAVEDSRFWELVDKERNLHLEGKCETCVYNMMGKREKKLGEFGKAKGSRAIWYMWL	477
NS51    NSAKEAVEDERFWDLVHRERELHKQGKCATCVYNMMGKREKKLGEFGKAKGSRAIWYMWL	477
NS53    DSARAAVEDEEFWKLVDRERELHKLGKCGSCVYNMMGKREKKLGEFGKAKGSRAIWYMWL	478
        **  **:*..**.**.:** **  *** :*****************:************

NS54    GARFLEFEALGFLNEDHWFGRENSWSGVEGEGLHRLGYILEEIDKKDGDLMYADDTAGWD	540
NS52    GARFLEFEALGFLNEDHWFSRENSLSGVEGEGLHKLGYILRDVSKKEGGAMYADDTAGWD	537
NS51    GARFLEFEALGFMNEDHWFSRENSLSGVEGEGLHKLGYILRDISKIPGGNMYADDTAGWD	537
NS53    GARYLEFEALGFLNEDHWFSRENSYSGVEGEGLHKLGYILRDISKIPGGAMYADDTAGWD	538
        ***:********:******.**** *********:*****.::.*  *. **********

NS54    TRITEDDLQNEELITEQMAPHHKILAKAIFKLTYQNKVVKVLRPTPRGAVMDIISRKDQR	600
NS52    TRITLEDLKNEEMVTNHMEGEHKKLAEAIFKLTYQNKVVRVQRPTPRGTVMDIISRRDQR	597
NS51    TRITEDDLQNEAKITDIMEPEHALLATSIFKLTYQNKVVRVQRPAKNGTVMDVISRRDQR	597
NS53    TRITEDDLHNEEKITQQMDPEHRQLANAIFKLTYQNKVVKVQRPTPKGTVMDIISRKDQR	598
        **** :**:**  :*: *  .*  ** :***********:* **: .*:***:***:***

NS54    GSGQVGTYGLNTFTNMEVQLIRQMEAEGVITQDDMQNPKGLKERVEKWLKECGVDRLKRM	660
NS52    GSGQVGTYGLNTFTNMEAQLIRQMEGEGVFKSIQHLTV-TEEIAVQNWLARVGRERLSRM	656
NS51    GSGQVGTYGLNTFTNMEAQLIRQMESEGIFSPSELETPNL-AERVLDWLEKYGVERLKRM	656
NS53    GSGQVGTYGLNTFTNMEAQLIRQMEGEGVLSKADLENPHPLEKKITQWLETKGVERLKRM	658
        *****************.*******.**::.  :  .       : .**   * :**.**

NS54    AISGDDCVVKPLDERFGTSLLFLNDMGKVRKDIPQWEPSKGWKNWQEVPFCSHHFHKIFM	720
NS52    AISGDDCVVKPLDDRFASALTALNDMGKVRKDIQQWEPSRGWNDWTQVPFCSHHFHELIM	716
NS51    AISGDDCVVKPIDDRFATALTALNDMGKVRKDIPQWEPSKGWNDWQQVPFCSHHFHQLIM	716
NS53    AISGDDCVVKPIDDRFANALLALNDMGKVRKDIPQWQPSKGWHDWQQVPFCSHHFHELIM	718
        ***********:*:**..:*  *********** **:**:**::* :*********:::*

NS54    KDGRSLVVPCRNQDELIGRARISQGAGWSLRETACLGKAYAQMWSLMYFHRRDLRLASMA	780
NS52    KDGRVLVVPCRNQDELIGRARISQGAGWSLRETACLGKSYAQMWSLMYFHRRDLRLAANA	776
NS51    KDGREIVVPCRNQDELVGRARVSQGAGWSLRETACLGKSYAQMWQLMYFHRRDLRLAANA	776
NS53    KDGRKLVVPCRPQDELIGRARISQGAGWSLRETACLGKAYAQMWTLMYFHRRDLRLASNA	778
        **** :***** ****:****:****************:***** ************: *

NS54    ICSAVPTEWFPTSRTTWSIHAHHQWMTTEDMLKVWNRVWIEDNPNMTDKTPVHSWEDIPY	840
NS52    ICSAVPSHWVPTSRTTWSIHAKHEWMTTEDMLTVWNRVWIQENPWMEDKTPVESWEEIPY	836
NS51    ICSAVPVDWVPTSRTTWSIHAHHQWMTTEDMLSVWNRVWIEENPWMEDKTHVSSWEDVPY	836
NS53    ICSAVPVHWVPTSRTTWSIHAHHQWMTTEDMLTVWNRVWIEDNPWMEDKTPVTTWEDVPY	838
        ****** .*.***********:*:********.*******::** * *** * :**::**

NS54    LGKREDLWCGSLIGLSSRATWAKNIHTAITQVRNLIGKEEYVDYMPVMKRYSAPSESEGV	900
NS52    LGKREDQWCGSLIGLTSRATWAKNIQTAINQVRSLIGNEEYTDYMPSMKRFRKEEEEAGV	896
NS51    LGKREDQWCGSLIGLTARATWATNIQVAINQVRRLIGNEEYLDYMTSMKRFKNESDPEGA	896
NS53    LGKREDQWCGSLIGLTSRATWAQNILTAIQQVRSLIGNEEFLDYMPSMKRFRKEEESEGA	898
        ****** ********::***** ** .** *** ***:**: ***  ***:   .:  *.

NS54    L-	901
NS52    LW	898
NS51    LW	898
NS53    IW	900
        :
```
